# Dynamics and turnover of memory CD8 T cell responses following yellow fever vaccination

**DOI:** 10.1371/journal.pcbi.1009468

**Published:** 2021-10-14

**Authors:** Veronika I. Zarnitsyna, Rama S. Akondy, Hasan Ahmed, Donald J. McGuire, Vladimir G. Zarnitsyn, Mia Moore, Philip L. F. Johnson, Rafi Ahmed, Kelvin W. Li, Marc K. Hellerstein, Rustom Antia

**Affiliations:** 1 Department of Microbiology and Immunology, Emory University, Atlanta, Georgia, United States of America; 2 Emory Vaccine Center, Emory University School of Medicine, Atlanta, Georgia, United States of America; 3 Trivedi School of Biosciences, Ashoka University, Sonipat, Haryana, India; 4 Department of Biology, Emory University, Atlanta, Georgia, United States of America; 5 Moonlight Therapeutics Inc., Atlanta, Georgia, United States of America; 6 Fred Hutchinson Cancer Research Center, Seattle, Washington, United States of America; 7 Department of Biology, University of Maryland, College Park, Maryland, United States of America; 8 Department of Nutritional Sciences and Toxicology, UC Berkeley, Berkeley, California, United States of America; Inria, FRANCE

## Abstract

Understanding how immunological memory lasts a lifetime requires quantifying changes in the number of memory cells as well as how their division and death rates change over time. We address these questions by using a statistically powerful mixed-effects differential equations framework to analyze data from two human studies that follow CD8 T cell responses to the yellow fever vaccine (YFV-17D). Models were first fit to the frequency of YFV-specific memory CD8 T cells and deuterium enrichment in those cells 42 days to 1 year post-vaccination. A different dataset, on the loss of YFV-specific CD8 T cells over three decades, was used to assess out of sample predictions of our models. The commonly used exponential and bi-exponential decline models performed relatively poorly. Models with the cell loss following a power law (exactly or approximately) were most predictive. Notably, using only the first year of data, these models accurately predicted T cell frequencies up to 30 years post-vaccination. Our analyses suggest that division rates of these cells drop and plateau at a low level (0.1% per day, ∼ double the estimated values for naive T cells) within one year following vaccination, whereas death rates continue to decline for much longer. Our results show that power laws can be predictive for T cell memory, a finding that may be useful for vaccine evaluation and epidemiological modeling. Moreover, since power laws asymptotically decline more slowly than any exponential decline, our results help explain the longevity of immune memory phenomenologically.

## Author summary

Immunological memory, generated in response to infection or vaccination, may provide complete or partial protection from antigenically similar infections for the lifetime. Memory CD8 T cells are important players in protection from secondary viral infections but quantitative understanding of their dynamics in humans is limited. We analyze data from two studies where immunization with the yellow fever virus vaccine (YFV-17D) generates a mild acute infection and long-term memory. We find that: (i) the division rate of YFV-17D-specific CD8 T cells drops and stabilizes at ∼ 0.1% per day during the first year following vaccination whereas the death rate declines more gradually, and (ii) the number of these cells declines approximately in accordance with a power law (∝ time^−0.75^) for at least several decades following vaccination.

## Introduction

Immunological memory is a central feature of the adaptive immune response that underlies successful vaccination, with memory CD8 T cells aiding in faster clearance of subsequent infections. How is the population of memory CD8 T cells maintained? In particular, how do division and death rates of expanded pathogen-specific CD8 T cells change with time after the pathogen is cleared? Can we predict the number of pathogen-specific cells decades after infection or vaccination based on the early immune response in humans? These are the key questions that we aim to address here.

Studies in mice following infections with viruses and bacteria, such as LCMV and Listeria monocytogenes, have greatly contributed to our understanding of immunological memory [1–8] (see also [9] for study in goats). Viral infections typically stimulate a rapid burst of proliferation of virus-specific CD8 T cells and the generation of a large population of effector cells that clear the virus. Subsequently, more than 90% of the virus-specific CD8 T cells die, and by day 30 post-infection there is a stable population of long-term memory cells [3–5, 8]. Turnover studies, first with bromodeoxyuridine (BrdU) [1, 2] and subsequently with carboxyfluorescein diacetate succinimidyl ester (CFSE) [6] showed that the memory cell population is maintained by the continuous turnover of cells with stochastic division (with an average time of about 50 days in mice) being balanced by death [6, 7].

Extending studies of immunological memory from mice (relatively short lifespans of 2–3 years) to humans (lifespans 70–100 years) has been particularly challenging. It has been shown that the overall number of virus-specific memory CD8 T cells after vaccination slowly declines through human life with an estimated half-life of T cell immunity of 8–15 years [10, 11]. Functional memory T cells can be detected for several decades after acute viral infection or immunization with live attenuated virus vaccines [12–14]. A number of studies have analyzed the turnover of bulk CD8 T cells in humans (typically based on deuterium incorporation) using different cell surface markers which have been associated with populations of naive, effector, memory and stem cell-like memory phenotypes [15–22]. A potential problem with this approach is the inability to unambiguously sort cells at different stages of T cell differentiation by using a few cell surface markers [23–25]. An alternative approach, and the one that we use in this paper, is to focus on a defined population of antigen-specific cells following immunization of individuals naive to the virus in the vaccine. We consider the merits and problems with these different approaches in the Discussion.

In this study, we analyze the dynamics and turnover of CD8 T cells following immunization of individuals with the live attenuated yellow fever vaccine (YFV-17D) [26], which generates a mild acute infection and confers long-term immunity. Our analysis integrates data from two studies. The study by Akondy et al. [24] (hereafter referred to as the Akondy study) *longitudinally* follows the frequencies and turnover of YFV-specific CD8 T cells in a group of 10 individuals during the first year after immunization with the YFV-17D vaccine. The frequency of CD8 T cells specific for an HLA-A2 restricted immunodominant epitope in the NS4B protein (A2-NS4B^214^) of the virus was determined using corresponding peptide-MHC class I tetramers (pMHCs) [23, 24]. The turnover of these cells was measured through labelling of their DNA following administration of heavy water (D_2_O) with subsequent analysis of the die-away kinetics of deuterium in the DNA of these cells after D_2_O administration was stopped. The study by Fuertes-Marraco et al. [14] (hereafter referred to as the Marraco study) was a *cross-sectional* survey that measured the frequency of YFV-specific T cells using A2-NS4B^214^ tetramers over a much longer time period ranging from a few months to three decades following YFV-17D vaccination.

We first build on the Akondy study for the dynamics and turnover of YFV-specific CD8 T cells during the early memory phase of the response. In particular, we quantify how the turnover rate of the YFV-specific CD8 T cell population declines over time and show that this decline is consistent with several models for changes in division and death rates of these cells. We then use the data from the Marraco study for model discrimination. Finally, we discuss how we can predict the frequency of virus-specific cells decades after vaccination using a power law model together with the data from the first year T cell responses.

## Results

### Dynamics of the YFV-response during the first year

**Fig 1:**
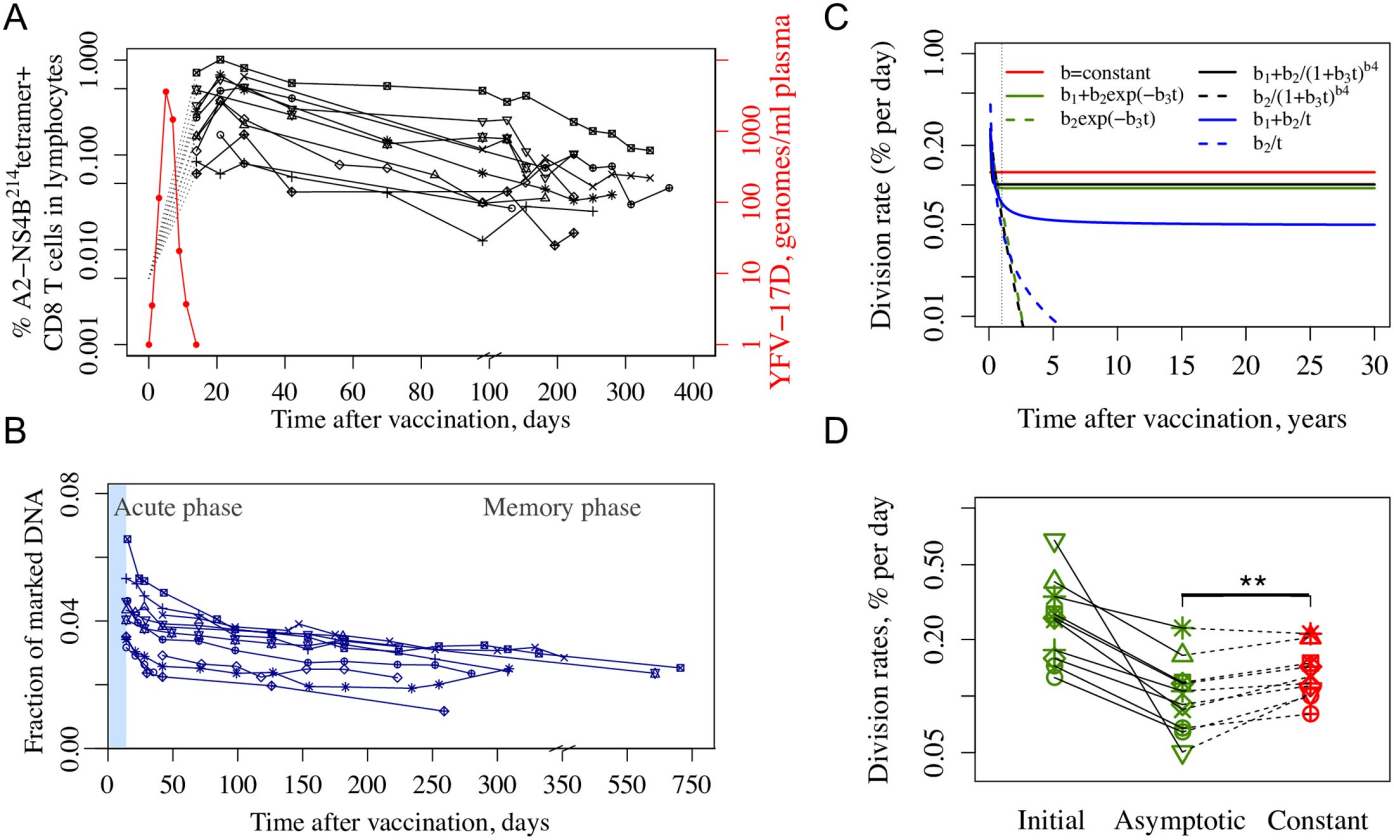
Dynamics and turnover of YFV-specific CD8 T cells in the first year after YFV-17D vaccination in the Akondy study. Panel A shows the dynamics of the virus (red) and YFV-specific CD8 T cell numbers (black) in the blood. Panel B shows the deuterium incorporation and die-away of label enrichment in YFV-specific CD8 T cells in individuals given D_2_O in their drinking water from day 0 to 14 post immunization (blue shaded region). Data in Panels A,B are from [24]. Panel C shows the estimated division rate over 30 years for all models tested (different functions describing the change of division rate over time are shown and color-coded as red, black, green and blue, see also fits to individual donors in S1 Fig). Panel D shows the division rate estimates with green symbols corresponding to initial and asymptotic division rates for the best model (*b*(*t*) = *b*_1_ + *b*_2_*exp*(−*b*_3_*t*)). The red symbols correspond to the division rates estimated from the constant division rate model. Each individual is shown by a different symbol. Solid and dotted lines connect the data for each individual obtained from the two models.

Immunization with YFV results in a mild acute infection with a peak of virus replication at around 5 to 7 days and total duration about 9 to 14 days (Fig 1A plots data from [24], also see [27] for details of the virus dynamics in different individuals). YFV-specific cells, measured using A2-NS4B^214^ tetramers, proliferate rapidly during the first 21–28 days post immunization, and subsequently their numbers gradually decline over time. The turnover of YFV-specific cells was determined using deuterium labelling. Individuals were given heavy water for the first two weeks following vaccination, which resulted in labelling of the DNA of dividing cells. After administration of deuterium labelled water was stopped, there was a rapid loss of D_2_O in serum (half-life of approximately 9 days [24]). The changes in the amount of deuterium in the DNA of YFV-specific cells are shown in Fig 1B.

Akondy et al. [24] analyzed the dynamics and turnover of YFV-specific cells during the early memory phase (from day 42 to ∼1 year post vaccination) by assuming constant rates for both the loss in cell numbers and the turnover of these cells. The estimated average rates for cell loss and rate of division of YFV-specific cells during this period were 0.57 ± 0.08% per day (half-life of about 122 days) and 0.15 ± 0.09% per day (cells divide on average once every 462 days) respectively. Here, we consider a more refined analysis allowing the rate of loss of YFV-specific cells and its underlying cell division and death rates to change over time. We also focus on the data from day 42 post immunization to analyze the memory phase. This choice simplified the calculations, since by this time there was negligible D_2_O in the serum allowing us to neglect further deuterium incorporation into the DNA of these dividing cells.

We expect that division and death rates initially decline and reach their asymptotic levels in the long run, and thus we considered several different time-dependent functions to capture this. The fitting to data was performed using a non-linear mixed effect modeling framework implemented in MonolixSuite 2020R1 (Lixoft).

We first estimated the division rate, *b*(*t*), from the data for the fraction of marked DNA (Fig 1B). Four different functional forms of *b*(*t*) were used to fit the data, and results are shown in Fig 1 Panels C,D and S1 and S2 Figs. In addition to a simple constant division rate (equivalent to the commonly used exponential decay model), the functional form *b*(*t*) = *b*_1_ + *b*_2_*exp*(−*b*_3_*t*) was chosen to allow division rate to have a relatively abrupt decline to the asymptote *b*_1_, while the functional form *b*(*t*) = *b*_1_ + *b*_2_/*t* was chosen to allow a more gradual decline. A general function $b\left(t\right)=b_{1}+b_{2}/{\left(1+b_{3}t\right)}^{b_{4}}$ allowed more flexibility with its parameter *b*_4_ allowing for either type of behavior. The models fits to the data were compared using the corrected Bayesian Information Criterion (BICc) (see Methods and [28]). The constant division rate model showed the worst fit (S1(A) and S2 Figs). Fitting the general function $b\left(t\right)=b_{1}+b_{2}/{\left(1+b_{3}t\right)}^{b_{4}}$ to the data returned a relatively large *b*_4_ = 10 with a major drop to a plateau level within a year after vaccination (solid black line in Fig 1C, S1(B)–S1(D) and S2 Figs). Interestingly, division rates estimated from the best fit model *b*(*t*) = *b*_1_ + *b*_2_*exp*(−*b*_3_*t*) and the general function model are very close at longer times (solid black and green lines in Fig 1C and S1(C) and S1(D) Fig). The asymptotic value *b*_1_ = 0.095 ± 0.024% per day (time between division of 730 days) of the best fit model is lower than 0.125 ± 0.018% per day (time between division of 555 days) estimated for the constant division rate model, and the difference is statistically significant (paired *t*-test *p*-value = 0.0035, Wilcoxon signed-rank test *p*-value = 0.0039 for green and red symbols in Fig 1D). The functional form *b*(*t*) = *b*_1_ + *b*_2_/*t*, with one less parameter than the best fit model and ΔBICc = 1, took longer to approach its asymptotic value of *b*_1_ = 0.049% per day. Note that the data do not support models, in which cells stop dividing in the long-term (version of the models with *b*_1_ = 0 and their corresponding ΔBICc are shown in parentheses in grey color in S1(A) Fig).

**Fig 2:**
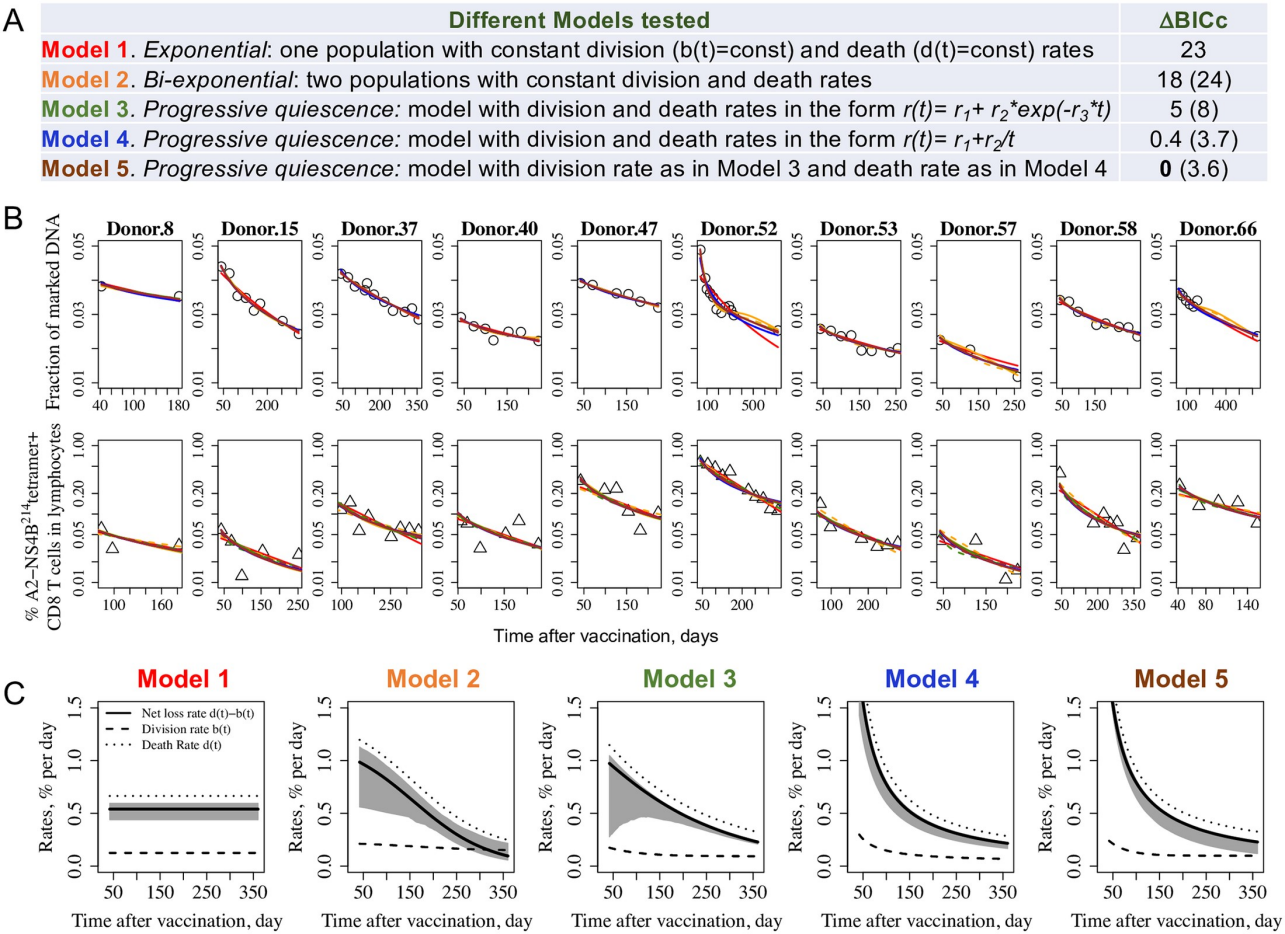
Models fit to changes in the tetramer positive YFV-specific CD8 T cells and their turnover over time. Panel A shows models describing different scenarios for changes in the division and death rates, b(t) and d(t), respectively, with time. In the simplest model (Model 1), the division and death rates are constant. Model 2 has two populations of cells with two different sets of constant division and death rates. In Models 3–5, the division and/or death rates can change from an initial value at day 42 to an asymptotic value long-term. Model 3 has both division and death rates as *r*(*t*) = *r*_1_ + *r*_2_*exp*(−*r*_3_*t*). Model 4 has both division and death rates modeled as *r*_1_ + *r*_2_/*t* and corresponds to a decline in number of T cells described by a power law, a functional form that captured the waning of antibodies to pertussis [29]. Model 5 has division rate as in Model 3 and death rate as in Model 4. We use a non-linear mixed effect modeling framework and the BICc information criteria [28] to examine how well these models fit the data from Akondy study [24, 30]. Models 2–5 have parameters restricted such that the asymptotic division and death rates equal each other (*restricted* models). ΔBICc values are shown for these models in relation to the best fit Model 5. ΔBICc for *unrestricted* versions of these models (long-term division and death rates are not restricted to be equal to each other) are shown in parentheses. Panel B shows how Models 1 (red line), Model 2 (orange line), Model 3 (green line), Model 4 (blue line), and Model 5 (brown line) capture both the change in the total number of cells (data—triangles) and the deuterium labelling (fraction of marked DNA, data—circles) for each individual. Solid lines correspond to the models with long-term division and death rates equal to each other and dashed lines correspond to the same models with unrestricted parameters (no requirement for long-term balancing in division and death rates). Panel C shows the predictions of models shown in Panel B for the division rate, death rate and overall net loss rate (i.e. death rate−division rate) and depicts the population average as obtained with the mixed effect approach. The 95% CI for the net loss rate is shaded in grey (see Methods section). Distribution of individual parameters for Models 1–5 are shown in S9–S13 Figs, and Visual Predictive Checks are shown in S7 and S8 Figs (see Methods).

The fraction of marked DNA data allowed us to estimate the division rates of YFV-specific CD8 T cells. In order to estimated the death rates, we next fitted both the data for the frequency of YFV-specific CD8 T cells and for the fraction of marked DNA (Fig 1A and 1B) simultaneously (see Methods). Similarly to the case of fitting the data for the fraction of marked DNA discussed above, we first used the model with the more general form (division rate $b\left(t\right)=b_{1}+b_{2}/{\left(1+b_{3}t\right)}^{b_{4}}$ and the same functional form for the death rate). Fitting this model to the data returned interesting observations: we inferred a relatively high value for parameter *b*_4_ for the division rate (>10) and a relatively low value for the corresponding parameter for the death rate *d*_4_ (<1), consistent with the division rate approaching its asymptotic level more abruptly than the death rate. BICc for this general form model was high in comparison to other models tested as expected due to the extra parameters, and it was excluded from further analysis. The results for the different models tested are shown in Fig 2. In the simplest model, the memoryless *exponential* model (Model 1, see Fig 2), the division and death rates are constants. Model 2 tests the hypothesis of the two populations with constant division and death rates: a “stem cell like” population that may expand early during the response but is relatively stable after day 42 with division and death rates equal to each other and another expanded population that is dying off with its *b* < *d*. Models 3–5 are different variations of the *progressive quiescence* model, where the overall population of YFV-specific cells progressively changes to establish long-term memory cells. Model 3 has both division and death rates change in the form *r*(*t*) = *r*_1_ + *r*_2_*exp*(−*r*_3_*t*), Model 4 has both division and death rates in the form *r*(*t*) = *r*_1_ + *r*_2_/*t*, and Model 5 has division rate as in Model 3 and death rate as in Model 4. For Models 2–5 we assume long-term division and death rates balance each other as suggested by mouse models [6, 7, 9], but also show how well the variants of Models 2–5 with unrestricted parameters (long-term division and death rates are not restricted to be equal each other) fit the data (ΔBICc numbers in parentheses in the right column in Fig 2A). Fig 2B shows how Models 1–5 capture both the change in the total number of cells and the fraction of marked DNA in YFV-specific cells for each individual. Individual model fittings for all donors are shown in S2 and S3 Figs. We note that the models capture the trend, but they are inaccurate for certain data points. This may reflect experimental noise or some complexity in dynamics that could not be captured by relatively simple models.

As foreseeable, the data do not support the *exponential* decline model with constant division and death rates (ΔBICc = 23 in comparison to the best fit Model 5). Although *bi-exponential* decline model (Model 2) with two populations each having constant division and death rates could fit the individual dynamics of the Akondy data alone, it also shows relatively poorer BICc in that context compared to Model 5 (ΔBICc = 18 for Model 2 with *b*_1_ = *d*_1_ and ΔBICc = 24 shown in parentheses for its *unrestricted* version).

Fig 2C shows the estimates for how the division and death rates and the difference between them (i.e. the net loss rate) change with time for each model (*restricted* Models 2–5). While changes in division rates occur in a relatively narrow range in all models, changes in death rates could be significant and still allow for the fitting of the Akondy data reasonably well. Thus, analysis of the data from the Akondy study alone did not allow us to distinguish between the three models with ΔBICc≤5, but these models make different predictions for the long-term loss of YFV-specific CD8 cells, and next we will bring these predictions into contact with the data in the Marraco study [14].

### Gradual loss of memory CD8 T cells allows model discrimination

**Fig 3:**
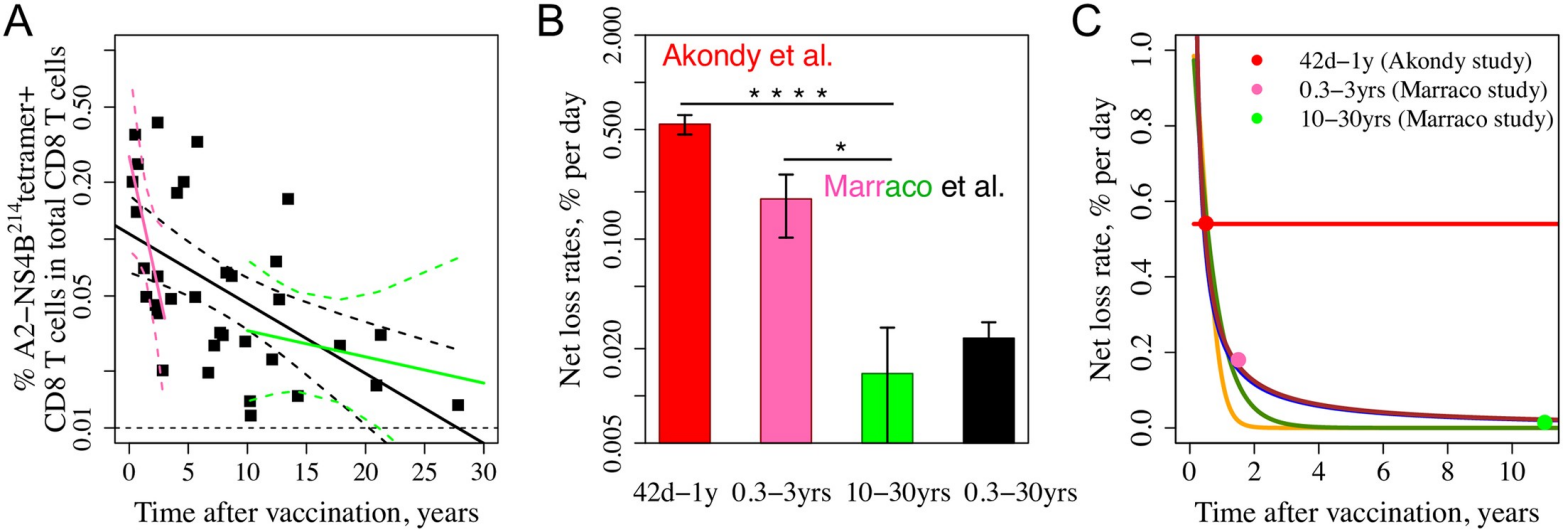
Dynamics of YFV-specific CD8 T cells over 30 years from the Fuertes-Marraco cross-sectional study [14]. Panel A shows the number of YFV-specific cells (on a log scale) as a function of time post vaccination. The slope gives the net rate of loss of the YFV-specific CD8 T cell response. The average rate of loss over the whole 30-year period is shown in the black line and the pink and green lines show the initial (first 3 years) and long-term (years 10–30) rates of cell loss, respectively. Dashed lines show respective 95% pointwise confidence intervals. Panel B summarizes the estimates from Panel A and compares them with the Akondy study. The net loss rate for the Akondy study was calculated by fitting a mixed effects model to tetramer data from day 42 and onwards assuming exponential decay (red bar). The mean and standard error values are shown. *p*-value < 1e-16 comparing the slope estimated in Akondy study and from 10–30 years in the Marraco study (flagged with four stars) and *p*-value = 0.03 comparing the slopes estimated from 0.3–3 years and 10–30 years in the Marraco study (flagged with one star) were calculated using Wald tests. Panel C shows the predictions from the Models 1–5 for the rate of cell loss, defined as the difference between the death and division rates at each time point, with Models 3–5 being more consistent with the experimental data. Filled circles are corresponding estimates from Panel B. Line colors: Model 1—red, Model 2—orange, Model 3—green, Model 4—blue, Model 5—brown. The blue and brown lines largely overlap.

The Marraco study [14] measured the numbers of YFV-specific T cells in individuals at different times during a period of 30 years following vaccination (Fig 3A). The black line shows the average decay rate for the entire dataset and gives a net loss of memory cells of 0.023% per day which corresponds to a “half-life” of about 8.25 years and is consistent with previous estimates [11]. Consistent with our analysis of the Akondy data, we find that the decay rate appears to decline over time. This can be seen by comparison of the pink line which indicates that for the first 3 years the rate of loss of memory cells is much faster (0.18% per day, half-life of only about 1 year) and the green line showing that after 10 years the estimated net loss rate is much lower (0.009% per day). Fig 3B compares these estimates with the Akondy study. The statistical significance for a slowing rate of decline using the Marraco data alone is not strong. For 0–3 years vs 10–30 years the *p*-value is only 0.03 (still significant, Fig 3B). 0–3 years was chosen for consistency with previously published studies [31–33], where 0–3 years was treated as a period of rapid waning. 10–30 years period was chosen to represent long-term memory. Notably, 0–3 years vs 3–30 years is also statistically significant (*p*-value = 0.041, Wald test), but 0–5 years vs 5–30 years and 0–10 years vs 10–30 years are not. Combined analysis of the Marraco and Akondy datasets results in strong statistical significance, e.g., the comparison between the Akondy data and the 10–30 years time frame of the Marraco data, as shown in Fig 3B, gives statistically significant *p*-value. This comparison remains strongly statistically significant (*p*-value < 1e-16) for other time frames of the Marraco data (e.g., 1–30 years, 3–30 years and 5–30 years).

We can bring predictions from our Models 1–5 into contact with the data in the cross-sectional Marraco study [14] in two different ways—by comparing (1) the net loss rate of the YFV-specific cells and (2) the number of YFV-specific cells over time. Fig 3C shows predictions for all models for the net loss rate (colored lines) together with estimates from the two experimental studies (circles, colors correspond to the bars in Fig 3B). Three models shown in green (Model 3), blue (Model 4) and brown (Model 5) color lines are consistent with the data. The predicted rate of loss of the YFV-specific cell population in these three models gradually falls over time, and a relatively stable memory cell population with a very low net loss rate is reached several years after immunization. The change in the rate of net loss of CD8 T cells is due to a slow decline of the death rate of the population over this time frame and only a modest, less than two fold, change in the division rate (see Fig 2C).

**Fig 4:**
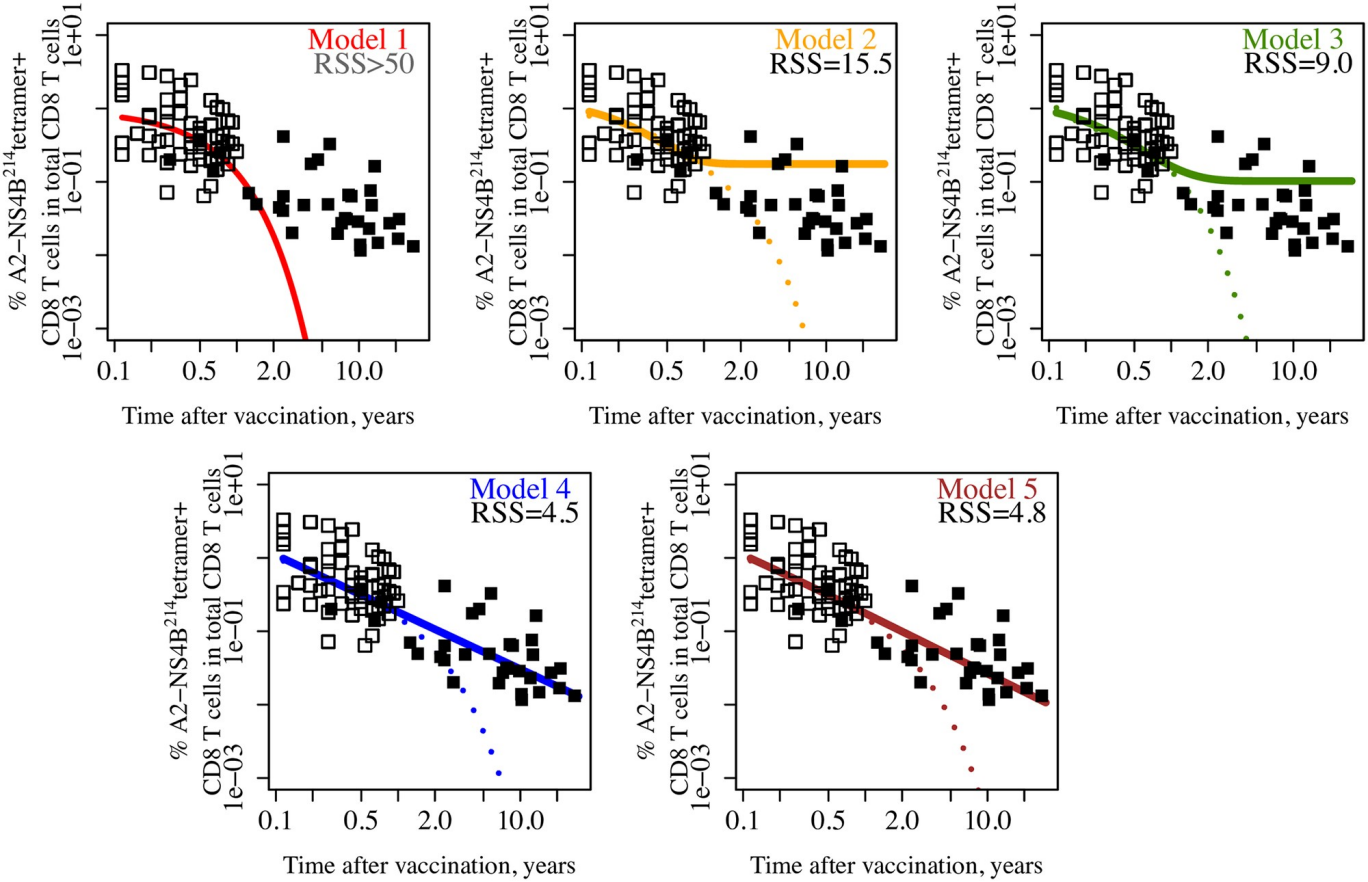
Integrated analysis of the data suggests that Models 4 and 5 predict the data in the Marraco study better than the other models. Data from the Akondy study [24] and the Marraco study [14] are shown as open and closed squares, respectively. For each model, we use fit to the Akondy data to predict the % of YFV-specific cells in total CD8 T cells up to 30 years after vaccination and compare this prediction with the Marraco data using residual sums of squares (RSSs). Dotted lines in panels with Model 2–5 show predictions from the corresponding models with unrestricted parameters. RSSs for these models are more than 50 (not shown) indicating a poor fit. As YFV-specific cells in the Akondy study were measured as % of lymphocytes and in the Marraco study as % of CD8 T cells, we converted the cell number predictions from fitting the Akondy data using the following formula. Since 45–70% of PBMCs are T cells and about 30% of them are CD8 T cells, we used a conversion factor equal to 0.575 ⋅ 0.3 ≈ 0.17. Plotting how this result depends on the conversion factor (S5 Fig) shows that Models 4 and 5 consistently show a better fit. Models colors are as in Fig 2: Model 1—red, Model 2—orange, Model 3—green, Model 4—blue, Model 5—brown.

Predictions of the models for a decline in the % of YFV-specific CD8 T cells together with the data from Akondy (open squares) and Marraco (filled squares) studies are shown in Fig 4. Residual sums of squares (RSSs) indicate how well the predictions fit the Marraco data. Model 4 and Model 5 fit data the best followed by Model 3 and then by Model 2. Model 2 has two populations both having constant division and death rates, and the division and death rates are equal to each other for one of the populations. Model 2 has a visually subtle but quantitatively worse fit to the Akondy data (ΔBICc = 18) as well as more poor predictions of the Marraco data (higher RSS). In Model 4, both division and death rates are in the form of *r*(*t*) = *r*_1_ + *r*_2_/*t*, while Model 5 uses *b*(*t*) = *b*_1_ + *b*_2_*exp*(−*b*_3_*t*) for the division rate and *d*(*t*) = *d*_1_ + *d*_2_/*t* for the death rate. While both Models 4 and 5 fit the Marraco’s data well and their RSS values are not significantly different (paired t-test on their squared residuals gives *p*-value = 0.87), they make different predictions regarding the long-term division rate. Model 4 predicts 0.037% per day (very slow doubling time ≈ 1863 days), while Model 5 predicts 0.098% per day (turnover rate ≈ 706 days). Model 5 may better describe the data overall as it is more consistent with the independent estimate of division rate as 0.15 ± 0.045% per day using data on D_2_O intake at 4–19 months post vaccination [24] (S6 Fig).

### Power law predicts change in YFV-specific cells

**Fig 5:**
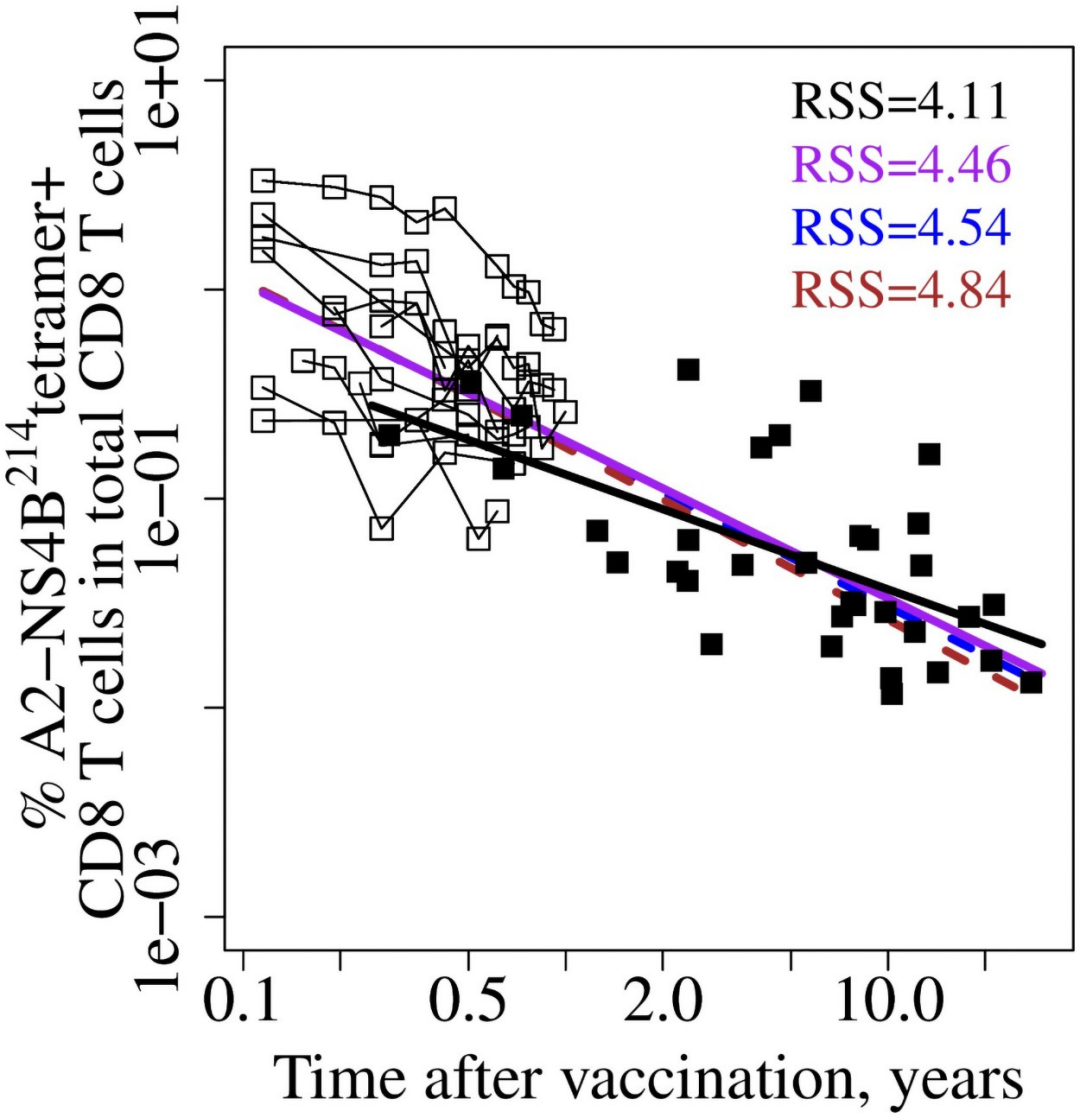
Predictions for Marraco’s data from Model 4 (blue line), Model 5 (brown line), and the simple power law model (purple line). Data from the Akondy study [24] and the Marraco study [14] are shown as open and closed squares, respectively. Data points for each individual donor in the Akondy study are connected by a thin black line. For Models 4 and 5, we fit the models to the Akondy data (Fig 1) from day 42 to 1 year using a mixed-effect framework to estimate the population parameters and based on these population parameters predict the frequency of YFV-specific cells up to 30 years after vaccination. We compare models predictions with the Marraco data using RSSs (RSS value colors matched the corresponding lines). Unlike Models 4 and 5, the simple power law model uses only YFV-specific T cells data (Fig 1A) and no DNA enrichment data. Use of a mixed-effect framework is essential for accurate prediction for all three models. Additionally, a power law fit for Marraco’s data only is shown with a black line.

Initial numbers and following changes in the net loss rate over time define the number of YFV-specific cells. Interestingly, the changes in the frequencies of YFV-specific cells in our two best models are described by power law functions *exactly* in Model 4 (division rate *b*(*t*) = 0.00037 + 0.11/*t*, death rate *d*(*t*) = 0.000372 + 0.89/*t*, so *b*(*t*) − *d*(*t*) = −0.78/*t* and, as *df*_*N*_/*dt* = (*b*(*t*) − *d*(*t*))*f*_*N*_, the solution of this ODE equation is frequency of YFV-specific cells *f*_*N*_(*t*) ∝ *t*^−0.78^ for t≥42 days) or *approximately* in Model 5 (*b*(*t*) = 0.00098 + 0.00425 ⋅ *e*^−0.0262*t*^, *d*(*t*) = 0.00098 + 0.823/*t*, so *b*(*t*) − *d*(*t*) ≈ −0.82/*t* as division rate *b*(*t*) ≈ 0.00098 after the first year, so *f*_*N*_(*t*) ≈ *c* ⋅ *t*^−0.82^ where *c* is a constant). We found that fitting longitudinal data in Fig 1A with a *simple power law* model from day 42 onwards using a mixed-effect framework can predict the number of YFV-specific cells for the decades after immunization. For this simple power law model we only use the frequencies of YFV-specific T cells and no DNA enrichment data (*df*_*N*_/*dt* = −*k*/*t* ⋅ *f*_*N*_, *f*_*N*_(*t*) ∝ *t*^−*k*^, *k* = 0.75). Fig 5 shows the simple power law model’s predictions from fitting the Akondy longitudinal data (purple line) in comparison to predictions from Models 4 (blue line) and Model 5 (brown line).

## Discussion

We developed a set of mathematical models to explore how the rates of division and death of YFV-specific CD8 T cells change with time after immunization with YFV-17D, a live-attenuated virus vaccine, that causes a mild acute infection. Analysis of the Akondy data alone allowed us to reject the commonly used exponential (ΔBICc = 23) and bi-exponential (ΔBICc = 18) decline models (Fig 2). Furthermore, there was poor support for models where the division rate asymptotes to zero in the long-term (S1 Fig) suggesting that long-term memory in humans is maintained by a population undergoing turnover. This result is qualitatively similar to memory cells in mice [1, 6], albeit the turnover of memory cells in mice (‘doubling time’ ≈50 days) is much faster than in humans (our estimated ‘doubling time’ ≈700 days). Combined analysis of the Akondy and Marraco data allowed us to discriminate between different models. Model 5, where division rate is in form *b*_1_ + *b*_2_*exp*(−*b*_3_*t*) and death rate is in form *d*(*t*) = *d*_1_ + *d*_2_/*t* and they asymptote to the same level (*b*_1_ = *d*_1_), albeit at different rates, has the best fit to the Akondy data, and fits the Marraco data almost as well as Model 4, but it is more consistent with the results of the study of D_2_O intake at 4–19 months post vaccination (S6 Fig). Models in which the frequency of YFV-specific cells follows a power law (exactly as in Model 4 or approximately from one year after vaccination onwards as in Model 5) are able to accurately predict the frequencies of YFV-specific CD8 T cells up to 30 years post vaccination.

An earlier study by Teunis et al. [29] suggested that the waning of antibodies to pertussis could be described better by a power law in comparison with exponential or bi-exponential forms of decay that were used earlier [31, 33, 34]. Our models suggest that the power law can also be used to describe the waning of CD8 T cell immunity. The gradual decline in the decay rate of CD8 T cell immunity is in line with previous studies for CD8 T cell memory following vaccinia immunization [10, 11].

There have been a number of earlier studies using deuterium labelling to quantify the turnover of T cells in humans [18, 19, 21, 22, 35–38]. Most of these studies identified CD8 memory cells on the basis of the CD45RO^+^ marker [18, 35, 38] and suggested that cells of this phenotype had a proliferation rate in the range of 0.6% to 2% per day [19, 35]. Further analysis suggested the disparity in these estimates could be reduced by using biphasic [36] or multi-exponential models [19], which suggests that there are subpopulations of CD45RO^+^ cells with different rates of turnover. Hellerstein et al. [36] analyzed bulk CD8 T-cell turnover in healthy subjects during longer-term labeling (between weeks 5–9) with D_2_O or longer term die-away (between weeks 3–7) after pulse labeling with 2H-glucose and reported 2% replacement per week by both approaches, or a division rate of 0.3% per day. More detailed phenotypic analysis of memory cells showed not only that there are different subpopulations of CD45RO^+^ memory cells, but also that at later times following immunization there are CD45RO^-^ memory cells which share many surface markers (CD45RO^-^, CCR7^+^, CD28^+^, CD127^+^) with naive CD8 T cells, but can be distinguished from naive cells on the basis of CXCR3, CD31, CD11a and CD95 [24, 39]. These cells have been termed stem-cell like memory cells or T_SCM_, and it has been shown that the fraction of cells with this phenotype increases with time following YFV immunization [24]. Consequently, the studies focusing on CD45RO^+^ cells will not describe the turnover of antigen-specific long-term memory CD8 T cells and they caution against attempts to identify a memory cell population with just a few surface markers.

Our estimate for the long-term division rate of YFV-specific cells is 0.1% per day and could be attributed to the T_SCM_ population, as a majority of cells detected decades after vaccination have a T_SCM_ phenotype [24]. This is substantially lower than a previous estimate of 0.6–7% per day [22] and positions the division rate for T_SCM_ cells between the rates for naive (0.03–0.06% per day [18, 22]) and memory (0.3–2% per day [19, 35, 36, 40]) cells.

There are caveats and limitations associated with our analysis, and we will now briefly discuss the important ones. As with many previous studies, we focus on data obtained from cells circulating in the blood and assume that the changes in the populations in the blood are representative of changes elsewhere. This is likely to be a reasonable assumption based on studies that demonstrate recirculation of memory T cells, but our analysis will not apply to resident memory populations that can play an important role in some infections [41]. A second limitation is that memory is associated with changes in both the number of antigen-specific CD8 T cells and a change in their phenotype, and this study focuses exclusively on the former. Recent studies have shown that CD8 T cells remain poised after primary response to have more rapid responses to repeat infections [8, 24], and the contribution to protection of phenotypic changes versus changes in numbers of antigen-specific cells has yet to be quantitatively evaluated. Finally, our studies consider turnover of the YFV-specific CD8 T cells as a whole—and at this stage lack of data precludes analysis of the differentiation pathways between different subpopulations which are likely to have different division and death rates. Deuterium labelling studies that follow cells of different phenotypes with a defined antigenic specificity will be needed to address this question in humans.

The immune system maintains immune memory and also responds to antigenically novel infections. Our results suggest that CD8 T cell memory declines approximately in accordance with a power law. In the case of the yellow fever vaccine, exponential decline at the rate observed 6 weeks to 1 year post vaccination (rate of cell loss defined as *b* − *d* and equal to 0.54% per day) would lead to dramatically fewer memory cells long term (>6 fold lower after only 10 years) compared to what is actually observed. Hence this exponential decline model is not only empirically inaccurate but potentially incompatible with longlasting protection via immune memory. In contrast, a power law is consistent with both the initial decline and long-term maintenance of memory CD8 T cells. Notably, a power law fit to the first year of data accurately predicted memory CD8 T cell numbers over three decades.

For new vaccines and emerging infections, such as SARS-CoV-2, where long term immune memory data is not yet available, power laws may be a useful alternative to exponential and biexponential models for predicting future waning of the immune response.

## Methods

### Derivation of the model equations

We consider the situation (day 42 and onwards after the vaccination) when virus has cleared and there is negligible D_2_O in the serum allowing us to neglect further deuterium incorporation into DNA of the cells [24]. Let us assume that *L*(*t*) is the total amount of deuterium incorporated in the YFV-specific CD8 T cells at time *t*. Note, that the division of cells does not change the total amount of labeled DNA, *L*(*t*), in the population of YFV-specific cells [42], but *L*(*t*) is gradually depleted by cell death. Under the assumption that (at a given time point) death rate is uncorrelated with the amount of deuterium in a cell, we can write:

$$\begin{array}{ccc} \;\frac{dL}{dt} & = & -d(t)L \end{array}$$ (1)

The total number of YFV-specific CD8 T cells, N, changes due to cellular division and death which occur at rates *b*(*t*) and *d*(*t*), respectively, and we can write:

$$\begin{array}{ccc} \;\frac{dN}{dt} & = & (b(t)-d(t))N \end{array}$$ (2)

Hence *f*_*L*_(*t*) = *L*(*t*)/*N*(*t*) is the average level of deuterium enrichment in YFV-specific cells at time *t*. Its change with time could be written as:

$$\begin{array}{c} \;\frac{df_{L}}{dt}=\frac{L^{{}^{\prime }}}{N}-\frac{LN^{{}^{\prime }}}{N^{2}}=-\frac{d(t)L}{N}-\left(b\left(t\right)-d\left(t\right)\right)\frac{L}{N}=-b\left(t\right)f_{L} \end{array}$$ (3)

and thus changes in *f*_*L*_ depend only on the division rate.

Assuming no change in the total number of virus-specific cells and that the constant division and death rates are balancing each other, our Eq (3) for the fraction of labeled DNA will convert to a previously proposed equation *df*_*L*_/*dt* = −*df*_*L*_, where *d* is a constant death rate (for review of different models with constant division and death rates see [43, 44]).

The formula for changes in *f*_*L*_ applies to Models 1, 3, 4 and 5. Similar reasoning yields a slightly different formula for Model 2 as it has two populations (see below).

### Mixed effects models

Mixed effects models were analyzed using MonolixSuite 2020R1 (Lixoft). As human data have high inter-individual variability and our data are sparse, we choose a mixed-effects modeling approach implemented in Monolix. The clear variability in our parameter estimates (supplemental S9–S13 Figs) validates this decision. The estimation of the population parameters was performed using the Stochastic Approximation Expectation-Maximization (SAEM) algorithm.

### Structural model

The Models 1, 3, 4 and 5 equations are (see derivation above):

$$\begin{array}{ccc} \text{(frequency}\;\text{of}\;\text{YFV-specific}\;\text{cells)}\;\frac{df_{N}}{dt} & = & \left(b\left(t\right)-d\left(t\right)\right)\cdot f_{N} \end{array}$$ (4)

$$\begin{array}{ccc} \text{(fraction}\;\text{of}\;\text{labeled}\;\text{DNA)}\;\frac{df_{L}}{dt} & = & -b\left(t\right)\cdot f_{L} \end{array}$$ (5)

We assume that total number of lymphocytes in the blood does not change with time and thus could normalized by it the number of YFV-specific cells *N* converting it to *f*_*N*_ and further multiply it by 100 to fit it to the data as % in lymphocytes.

We assume that both division rate *b*(*t*) and death rate *d*(*t*) change with time and asymptote to non-zero values, unless indicated otherwise. The asymptotic value of the division rate was used to calculate ‘doubling time’ as *ln*(2)/*b*. Functions used to model division and death rates are shown in Fig 2A and S1(A) Fig. In Figs 2B and 4 we plot two versions for Models 2–5. *“Restricted”* means that asymptotic division and death rates are equal to each other. *“Unrestricted”* means that for the corresponding parameters (*b*_1_ and *d*_1_) there is no requirement for long-term balancing in division and death rates. Since we focus on fitting the data from day 42 post immunization (28 days after administration of deuterium labelled water was stopped), the heavy water had largely washed out (half-life of approximately 9 days [24]) allowing us to neglect further deuterium incorporation into DNA of the cells and to simplify the analysis.

For the two-population bi-exponential decline model (Model 2), we used two sets of equations each similar to the one population exponential decline model and fit the data shown in Fig 1A and 1B with *f*_*N*_ = *f*_*N*1_ + *f*_*N*2_ and *f*_*L*_ = (*f*_*L*1_ ⋅ *f*_*N*1_ + *f*_*L*2_ ⋅ *f*_*N*2_)/(*f*_*N*1_ + *f*_*N*2_), where subscripts correspond to these two populations.

For Fig 5, we fit the Akondy longitudinal data for frequency of YFV-specific cells with the equation *df*_*N*_/*dt* = −*k*/*t* ⋅ *f*_*N*_ (for the simple power law model) using Monolix. We fit the Marraco data using log-log linear regression in *R* using the *lm*() function.

### Individual model

We assume that individual-level parameters are lognormally distributed as *log*(*θ*_*i*_) = *log*(*θ*_*pop*_) + *η*_*i*_ with *η*_*i*_ ∼ *N*(0, Σ).

Here *i* is the index for individuals, *θ*_*i*_ is a vector of of individual i’s model parameters, *θ*_*pop*_ is the population-level fixed effects and *η*_*i*_ is the individual-level random effects. We assume no correlations between the random effects (i.e. the covariance matrix Σ is diagonal). For parameters characterizing death rate, only fixed effects were used. Inclusion of random effects for the death rate parameters led to a high error in estimating the corresponding variances. Likewise, for the tetramer only model used to fit data in Fig 5, we assumed random effects only for YFV-specific cells values at day 42, but not for the parameter describing decay rate.

For plotting individual fits (Fig 2B and S2–S4 Figs) and individual parameters (S9–S13 Figs), we used the posterior mode of the random effects for each individual. For individual *i* the posterior mode is the value of *η*_*i*_ that maximizes $f\left(y_{i}|\eta_{i},{\hat{\theta }}_{pop},\hat{\sigma }\right)\cdot f\left(\eta_{i}|\hat{\sum }\right)$ where *f* signifies probability density, *y*_*i*_ is the observed data for individual *i* and ${\hat{\theta }}_{pop}$, $\hat{\sigma }$ and $\hat{\sum }$ are the maximum likelihood estimates for the fixed effects, the residual standard deviation and the random effects covariance matrix, respectively. Note that the mixed effects likelihood

$$\begin{array}{c} \int f\left(y|\eta ,{\hat{\theta }}_{pop},\hat{\sigma }\right)\cdot f\left(\eta |\hat{\sum }\right)\;d\eta \end{array}$$ (6)

involves integrating over all values of the random effects. Hence, in contrast to fixed effects, the accuracy of the individual fits (at the posterior modes) is not always reflective of the model accuracy.

### Residual error model

For the fraction of labeled DNA, we assumed additive independent (mean = 0) Gaussian observation error. For the frequency of YFV-specific CD8 T cells we assumed multiplicative independent (median = 1) lognormal observation error.

### Data inclusion criteria

We analyzed the dynamics and turnover of CD8 T cells following immunization of individuals with the live attenuated yellow fever vaccine using data shown in Fig 1A and 1B. As we focused on the memory phase, we used only the data starting from day 42 post immunization and onwards. The criterion for inclusion of a donor in the analysis was to have two or more time points for both the frequency of the YFV-specific CD8 T cells and DNA enrichment. One time point (t = 309 days for DNA enrichment for donor 53) was excluded from the analysis. Inclusion of this point in the analysis marked it as a clear outlier (e.g. on graphs evaluating observations versus predictions). The fraction of labeled DNA went up from 0.02 at the previous time point t = 255 days to 0.025 at t = 309 days.

To plot the data from Marraco study in Figs 3–5, we digitized the corresponding data from Fig 1B in the Fuertes-Marraco cross-sectional study [14] using WebPlotDigitizer (https://apps.automeris.io/wpd/).

### Model discrimination

Maximum likelihood was used to fit both the deuterium enrichment and frequency of YFV-specific CD8 T cells in the Akondy study. A new corrected Bayesian Information Criterion (BICc) [28], implemented in Monolix, was used to compare the different models. The criterion is similar to BIC but defines sample size in a way that is more tailored for mixed effects models. To additionally evaluate how well a given model can reproduce both the main trend and the variability in the data, we used Visual Predictive Check implemented in Monolix. The corresponding plots in S7 and S8 Figs compare the theoretical percentiles (computed from multiple Monte Carlo simulations) with empirical percentiles of the observed data for Models 1–5. To add 95% CIs in Fig 2C and S6(B) Fig, we ran 1000 simulations for each model with Simulx (Lixoft, France) using the corresponding population parameters obtained from fitting the data in Monolix.

## Supporting information

S1 FigDivision rate only models fit to the fraction of marked DNA shown in Fig 1B.Panel A shows the Table with different functions describing the change of division rate over time in different models color-coded as red (division rate *b*(*t*) = constant), black (general form $b\left(t\right)=b_{1}+b_{2}/{\left(1+b_{3}t\right)}^{b_{4}}$), green (*b*(*t*) = *b*_1_ + *b*_2_*exp*(−*b*_3_*t*)) and blue (*b*(*t*) = *b*_1_ + *b*_2_/*t*). Corresponding ΔBICc are listed in comparison to the best model *b*(*t*) = *b*_1_ + *b*_2_*exp*(−*b*_3_*t*). Cases where long-term division rate is equal to zero (*b*_1_ = 0) showed the same or worse fit for all functional forms as shown by ΔBICc in the brackets with grey color for each model. Panel B shows the fitting results for individual donors using data from day 42 upward for each model with colors as in Panel A. Panels C and D show the estimated division rate during the first year and long-term over 30 years for all models tested. Vertical dotted line in Panel D corresponds to 1 year.(TIFF)

S2 FigIndividual fits from the division rate only models in S1 Fig.Different functions (see S1(A) Fig) describing the change in division rate over time in different models color-coded as red (division rate *b*(*t*) = constant), black (general form $b\left(t\right)=b_{1}+b_{2}/{\left(1+b_{3}t\right)}^{b_{4}}$), green (*b*(*t*) = *b*_1_ + *b*_2_*exp*(−*b*_3_*t*)) and blue (*b*(*t*) = *b*_1_ + *b*_2_/*t*)).(TIFF)

S3 Fig Individual fits for the deuterium labelling in Akondy data from models described in Fig 2. Individual panels show how Models 1 (red line), Model 2 (orange line), Model 3 (green line), Model 4 (blue line), and Model 5 (brown line) capture the deuterium labelling (fraction of marked DNA, data—circles) for each donor. In the simplest model (Model 1), the division and death rates are constant. Model 2 has two populations of cells with two different sets of constant division and death rates. In Models 3–5, the division and/or death rates can change from an initial value at day 42 to an asymptotic value long-term. Model 3 has both division and death rates as *r*(*t*) = *r*_1_ + *r*_2_*exp*(−*r*_3_*t*). Model 4 has both division and death rates modeled as *r*_1_ + *r*_2_/*t*. Model 5 has division rate as in Model 3 and death rate as in Model 4. We use a non-linear mixed effect modeling framework implemented in Monolix. Solid lines correspond to the models with long-term division and death rates equal each other and dashed lines correspond to the same models with unrestricted parameters (no requirement for long-term balancing in division and death rates).
(TIFF)

S4 Fig Individual fits for the frequencies of YFV-specific CD8 T cells in Akondy data shown in Fig 1A. Individual panels show how Model 1 (red line), Model 2 (orange line), Model 3 (green line), Model 4 (blue line), and Model 5 (brown line) capture the % of YFV-specific CD8 T cells in total CD8 T cells, data—triangles) for each Donor. In the simplest model (Model 1), the division and death rates are constant. Model 2 has two populations of cells with two different sets of constant division and death rates. In Models 3–5, the division and/or death rates can change from an initial value at day 42 to an asymptotic value long-term. Model 3 has both division and death rates as *r*(*t*) = *r*_1_ + *r*_2_*exp*(−*r*_3_*t*). Model 4 has both division and death rates modeled as *r*_1_ + *r*_2_/*t*. Model 5 has division rate as in Model 3 and death rate as in Model 4. We use a non-linear mixed effect modeling framework implemented in Monolix. Solid lines correspond to the models with long-term division and death rates equal each other and dashed lines correspond to the same models with unrestricted parameters (no requirement for long-term balancing in division and death rates).
(TIFF)

S5 Fig Dependence of the residual sums of squares (RSS) for Models 2–5 on the conversion factor. YFV-specific cells in the Akondy study were measured as % in lymphocytes and in the Marraco study as % in CD8 T cells, thus we converted the cell numbers from fitting Akondy data and model predictions using the following reasonings. Since 45–70% of PBMCs are T cells and about 30% of them are CD8 T cells, we used a conversion factor equal to 0.575 ⋅ 0.3 ≈ 0.17 in Fig 4. Plotting how this result depends on the conversion factor in a range of between 0.135 and 0.21 shows that Models 4 and 5 consistently show a better fit. Models colors are as in Fig 2: Model 2—orange, Model 3—green, Model 4—blue, Model 5—brown. The conversion factor only affects how well the predictions of the Models 1–5 will fit the Marraco data based on RSS. Thus, conversion factor will not affect the estimation of the CD8 T cell lifespans.
(TIFF)

S6 Fig Predictions from Models 4 and 5 compared to estimate for deuterium incorporation at later memory stage. Panel A shows deuterium incorporation in six donors with heavy water consumption for 8 weeks starting at 4–19 months after YFV immunization (Study 3 in [24]). Data for different individuals are shown by different symbols. Pink shaded area indicates the period of heavy water consumption for each donor. Panel B shows how the predicted division rates (with their 95% confidence intervals (CI)) change with time. The asymptotic division rate for Model 4 (blue line) was estimated as 0.037% per day (95% CI 0.011–0.16) and for Model 5 (brown line) as 0.1% per day (95% CI 0.04139–0.236). The study analyzed the deuterium incorporation at the memory stage shown in Panel A [24], and the corresponding estimate for division rate, equal to 0.15±0.045% per day, is shown by a rectangle with 4–19 month length on the x-axis and standard deviation estimation on y-axis.
(TIFF)

S7 Fig Visual Predictive Check (VPC) plots for fitting fraction of marked DNA (data in Fig 1B) using Models 1–5 described in Fig 2A. To additionally evaluate how well a given model can reproduce both the main trend and the variability in the data, we used Visual Predictive Check as implemented in Monolix. Different percentiles of the observed data (blue lines show 10th, 50th and 90th percentiles) are compared to 90% prediction intervals for those percentiles according to the models (blue shaded regions for 10th and 90th percentiles, pink for 50th percentile and purple for overlap). Red circles around a point indicate mismatch between the empirical percentiles and the model. Data are grouped together within bins of an independent variable (time). Binning criterion was “least-squares” (as implemented in Monolix) and bins are shown by red lines. VPC does not account for dropout. Most individuals dropped out before day 300 and hence VPC is not valid for the last bin shown in this figure.
(TIFF)

S8 Fig Visual Predictive Check (VPC) plots for fitting frequencies of YFV-specific cells (data in Fig 1A) using Models 1–5 described in Fig 2A. To additionally evaluate how well a given model can reproduce both the main trend and the variability in the data, we used Visual Predictive Check as implemented in Monolix. Different percentiles of the observed data (blue lines show 10th, 50th and 90th percentiles) are compared to 90% prediction intervals for those percentiles according to the models (blue shaded regions for 10th and 90th percentiles, pink for 50th percentile and purple for overlap). Red circles around a point indicate mismatch between the empirical percentiles and the model. Data are grouped together within bins of an independent variable (time). Binning criterion was “least-squares” (as implemented in Monolix) and bins are shown by red lines.
(TIFF)

S9 FigDistribution of individual parameters in Model 1.FL42 and FN42 are estimated *f*_*L*_ and *f*_*N*_ values at day 42, and b is a constant division rate. The death rate parameter d = 0.0067 per day (s.e. = 0.0007 per day; no random effects). Shrinkage for a model parameter *η* is calculated as $1-var\left(\hat{\eta_{i}}\right)/{\hat{\omega }}^{2}$, where $\hat{\eta_{i}}$ are the posterior modes and $\hat{\omega }$ is the estimated standard deviation of the random effects.(TIFF)

S10 FigDistribution of individual parameters in Model 2.FL42 and FN42 are estimated *f*_*L*_ and *f*_*N*_ values at day 42. Parameters b1 and b2 are constant division rates for the two populations and other parameters are described in Methods. The death rate parameters are d1 = b1 and d2 = 0.0145 per day (s.e. = 0.0014 per day; no random effects). Shrinkage for a model parameter *η* is calculated as $1-var\left(\hat{\eta_{i}}\right)/{\hat{\omega }}^{2}$, where $\hat{\eta_{i}}$ are the posterior modes and $\hat{\omega }$ is the estimated standard deviation of the random effects.(TIFF)

S11 FigDistribution of individual parameters in Model 3.FL0 and FN0 are estimated *f*_*L*_ and *f*_*N*_ values at day 42. Parameters kb1, kb2, kb3 are estimates for the division rate in b(t) = kb1+kb2 exp(-kb3 t). The death rate parameters are d1 = kb1, d2 = 0.0129 per day (s.e. = 0.0017 per day) and d3 = 0.0048 (s.e. = 0.0004) in d(t) = d1+d2 exp(-d3 t) (no random effects for d2 and d3). Shrinkage for a model parameter *η* is calculated as $1-var\left(\hat{\eta_{i}}\right)/{\hat{\omega }}^{2}$, where $\hat{\eta_{i}}$ are the posterior modes and $\hat{\omega }$ is the estimated standard deviation of the random effects.(TIFF)

S12 FigDistribution of individual parameters in Model 4.FL0 and FN0 are estimated *f*_*L*_ and *f*_*N*_ values at day 42. Parameters kb1, kb2 are estimates for the division rate in b(t) = kb1+kb2/t. The death rate parameters are d1 = kb1, d2 = 0.887 (s.e. = 0.0103) in d(t) = d1+d2/t (no random effects for d2). Shrinkage for a model parameter *η* is calculated as $1-var\left(\hat{\eta_{i}}\right)/{\hat{\omega }}^{2}$, where $\hat{\eta_{i}}$ are the posterior modes and $\hat{\omega }$ is the estimated standard deviation of the random effects.(TIFF)

S13 FigDistribution of individual parameters in Model 5.FL0 and FN0 are estimated *f*_*L*_ and *f*_*N*_ values at day 42. Parameters kb1, kb2, kb3 are estimates for the division rate in b(t) = kb1+kb2 exp(-kb3 t). The death rate parameters are d1 = kb1, d2 = 0.823 (s.e = 0.0186) in d(t) = d1+d2/t (no random effects for d2). Shrinkage for a model parameter *η* is calculated as $1-var\left(\hat{\eta_{i}}\right)/{\hat{\omega }}^{2}$, where $\hat{\eta_{i}}$ are the posterior modes and $\hat{\omega }$ is the estimated standard deviation of the random effects.(TIFF)

S1 Code The archived folder with experimental data and R code to generate the manuscript figures (FilesForRCode.zip) and the results of model fitting to data using MonolixSuite 2020R1 (MonolixResults.zip). The archived folder FilesForRCode.zip includes the files described below. CompleteData.csv has data from the Akondy study for Fig 1A and 1B. DNAEnrichmentDay42up.csv and YFTcellsinLymphocytesD42up.csv have the subsets of the data from CompleteData.csv to model the fraction of marked DNA and the frequency of YFV-specific cells from day 42 onwards. MarracoFigure1B.csv has digitized data from the Marraco study. DNAenrichmentStudy3.csv has data for S6 Fig. The files ModelParameters.csv, MonolixFitForFigure2B.csv and MonolixFitForSupplementalFigure1B.csv have the organized data from the models fitting for the corresponding figures. Figure2CFiles folder has files for generating Fig 2C with Simulx. YFpaperCodeForFigures.Rmd has the R code to generate the figures in the paper. The archived folder MonolixResults.zip includes the files described below. Four subfolders have the corresponding models and fits for Fig 1C and 1D, S1 and S2 Figs (DNAErnichmentOnly folder), Fig 3B (TetramersOnlyFigure3BAkondy folder), Figs 2, 3C and 4 (AkondyStudy folder), and purple line in Fig 5 (TetramersOnlyFigure5 folder).
(ZIP)
