# Supplementary figures and images for: Dynamics and turnover of memory CD8 T cell responses following yellow fever vaccination
Source: PLoS Comput Biol. 2021 Oct 14;17(10):e1009468. doi: 10.1371/journal.pcbi.1009468 (PMC8568194; doi:10.1371/journal.pcbi.1009468)

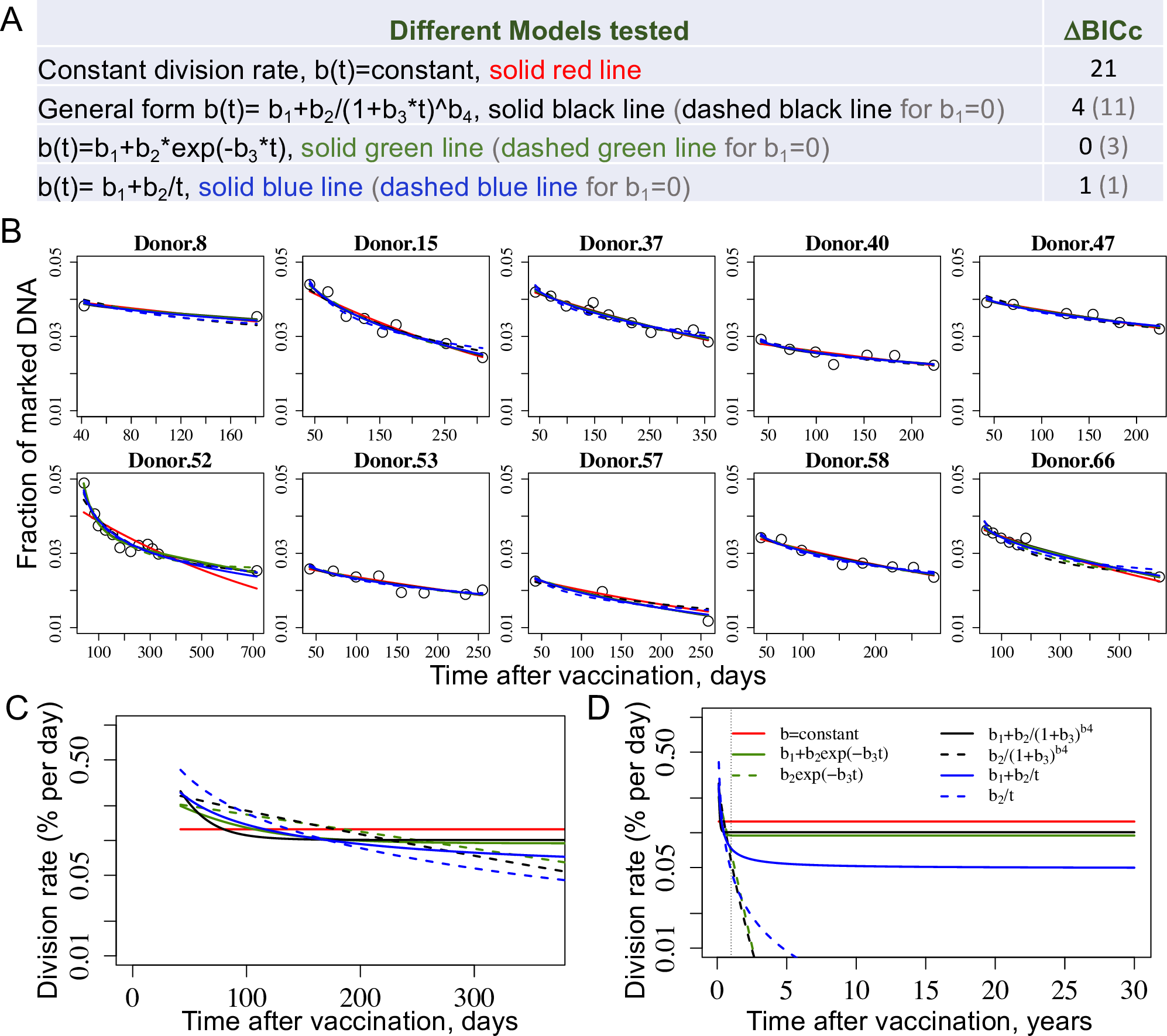

Supplement: S1 Fig — Panel A shows the Table with different functions describing the change of division rate over time in different models color-coded as red (division rate b(t) = constant), black (general form b(t)=b1+b2/(1+b3t)b4), green (b(t) = b1 + b2exp(−b3t)) and blue (b(t) = b1 + b2/t). Corresponding ΔBICc are listed in comparison to the best model b(t) = b1 + b2exp(−b3t). Cases where long-term division rate is equal to zero (b1 = 0) showed the same or worse fit for all functional forms as shown by ΔBICc in the brackets with grey color for each model. Panel B shows the fitting results for individual donors using data from day 42 upward for each model with colors as in Panel A. Panels C and D show the estimated division rate during the first year and long-term over 30 years for all models tested. Vertical dotted line in Panel D corresponds to 1 year. (TIFF) [file pcbi.1009468.s001.tiff]

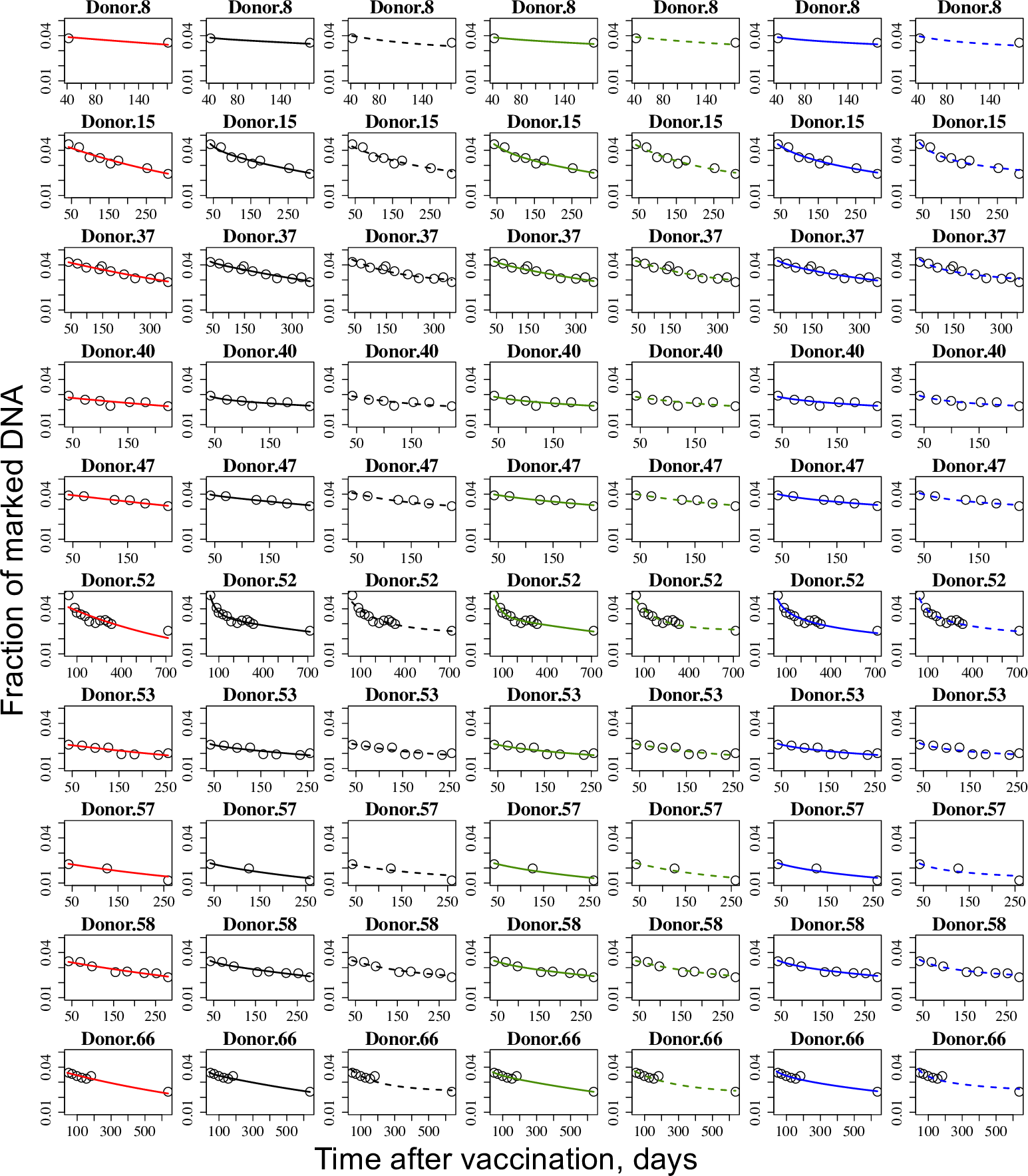

Supplement: S2 Fig — Different functions (see S1(A) Fig) describing the change in division rate over time in different models color-coded as red (division rate b(t) = constant), black (general form b(t)=b1+b2/(1+b3t)b4), green (b(t) = b1 + b2exp(−b3t)) and blue (b(t) = b1 + b2/t)). (TIFF) [file pcbi.1009468.s002.tiff]

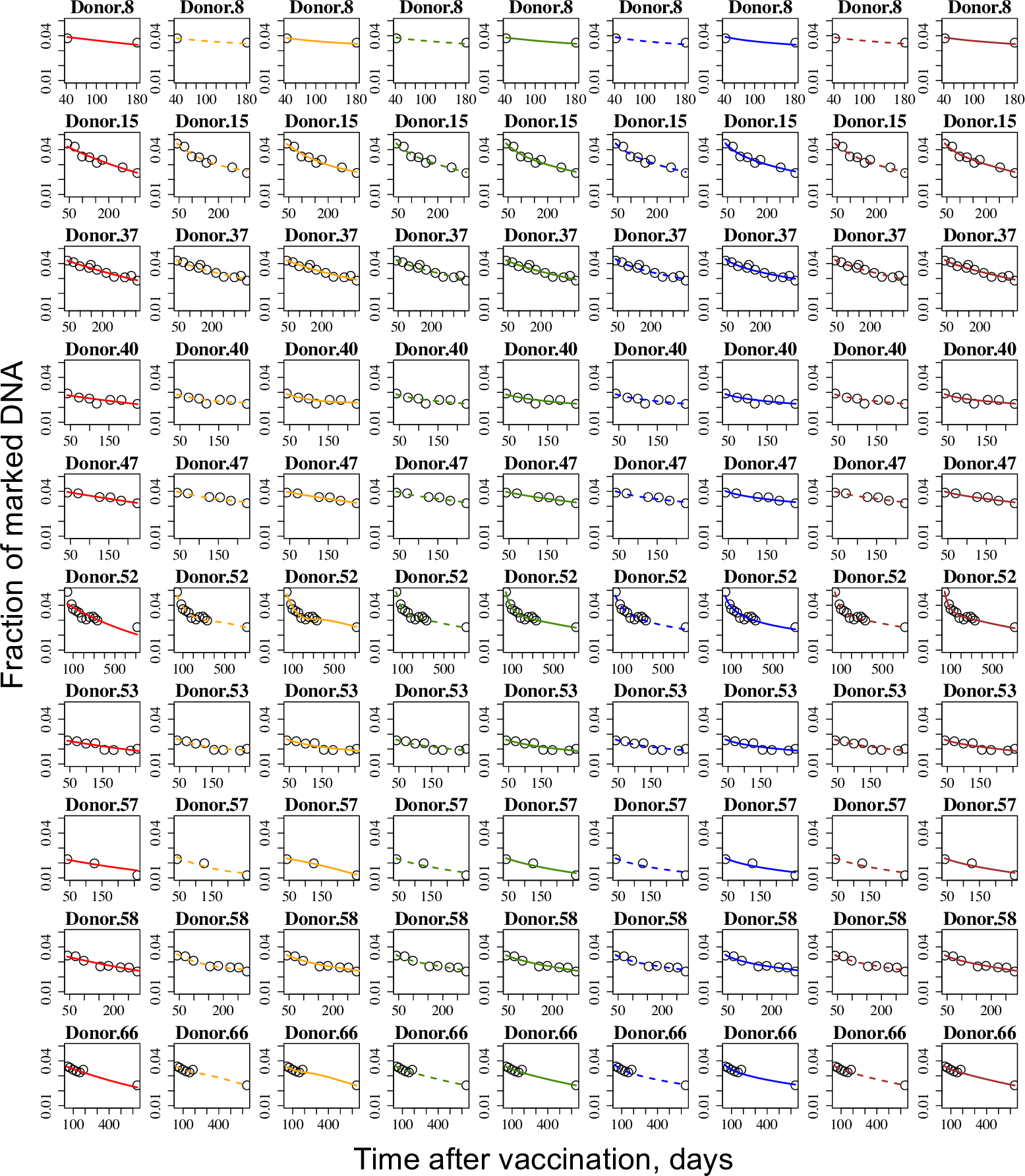

Supplement: S3 Fig — Individual panels show how Models 1 (red line), Model 2 (orange line), Model 3 (green line), Model 4 (blue line), and Model 5 (brown line) capture the deuterium labelling (fraction of marked DNA, data—circles) for each donor. In the simplest model (Model 1), the division and death rates are constant. Model 2 has two populations of cells with two different sets of constant division and death rates. In Models 3–5, the division and/or death rates can change from an initial value at day 42 to an asymptotic value long-term. Model 3 has both division and death rates as r(t) = r1 + r2exp(−r3t). Model 4 has both division and death rates modeled as r1 + r2/t. Model 5 has division rate as in Model 3 and death rate as in Model 4. We use a non-linear mixed effect modeling framework implemented in Monolix. Solid lines correspond to the models with long-term division and death rates equal each other and dashed lines correspond to the same models with unrestricted parameters (no requirement for long-term balancing in division and death rates). (TIFF) [file pcbi.1009468.s003.tiff]

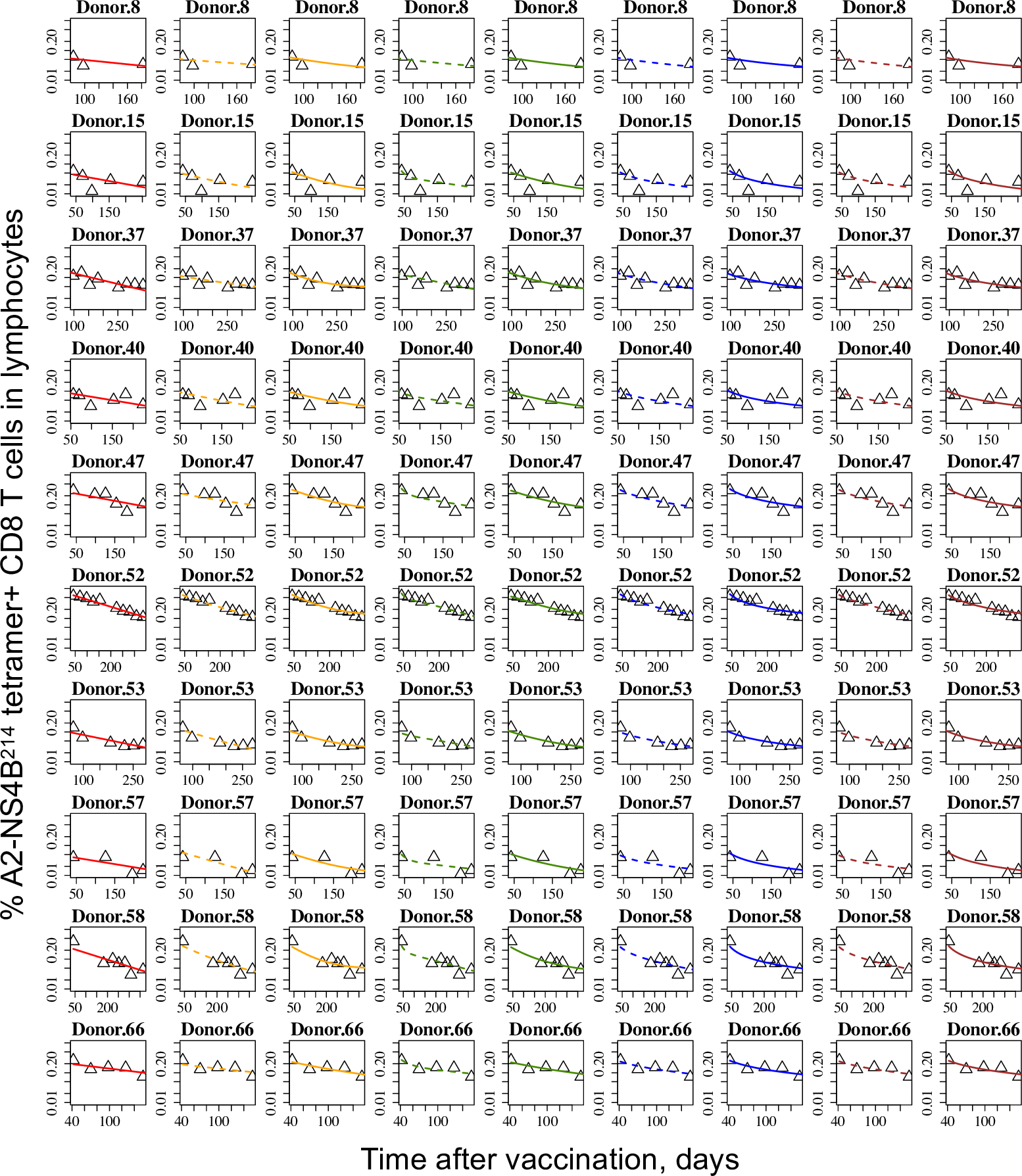

Supplement: S4 Fig — Individual panels show how Model 1 (red line), Model 2 (orange line), Model 3 (green line), Model 4 (blue line), and Model 5 (brown line) capture the % of YFV-specific CD8 T cells in total CD8 T cells, data—triangles) for each Donor. In the simplest model (Model 1), the division and death rates are constant. Model 2 has two populations of cells with two different sets of constant division and death rates. In Models 3–5, the division and/or death rates can change from an initial value at day 42 to an asymptotic value long-term. Model 3 has both division and death rates as r(t) = r1 + r2exp(−r3t). Model 4 has both division and death rates modeled as r1 + r2/t. Model 5 has division rate as in Model 3 and death rate as in Model 4. We use a non-linear mixed effect modeling framework implemented in Monolix. Solid lines correspond to the models with long-term division and death rates equal each other and dashed lines correspond to the same models with unrestricted parameters (no requirement for long-term balancing in division and death rates). (TIFF) [file pcbi.1009468.s004.tiff]

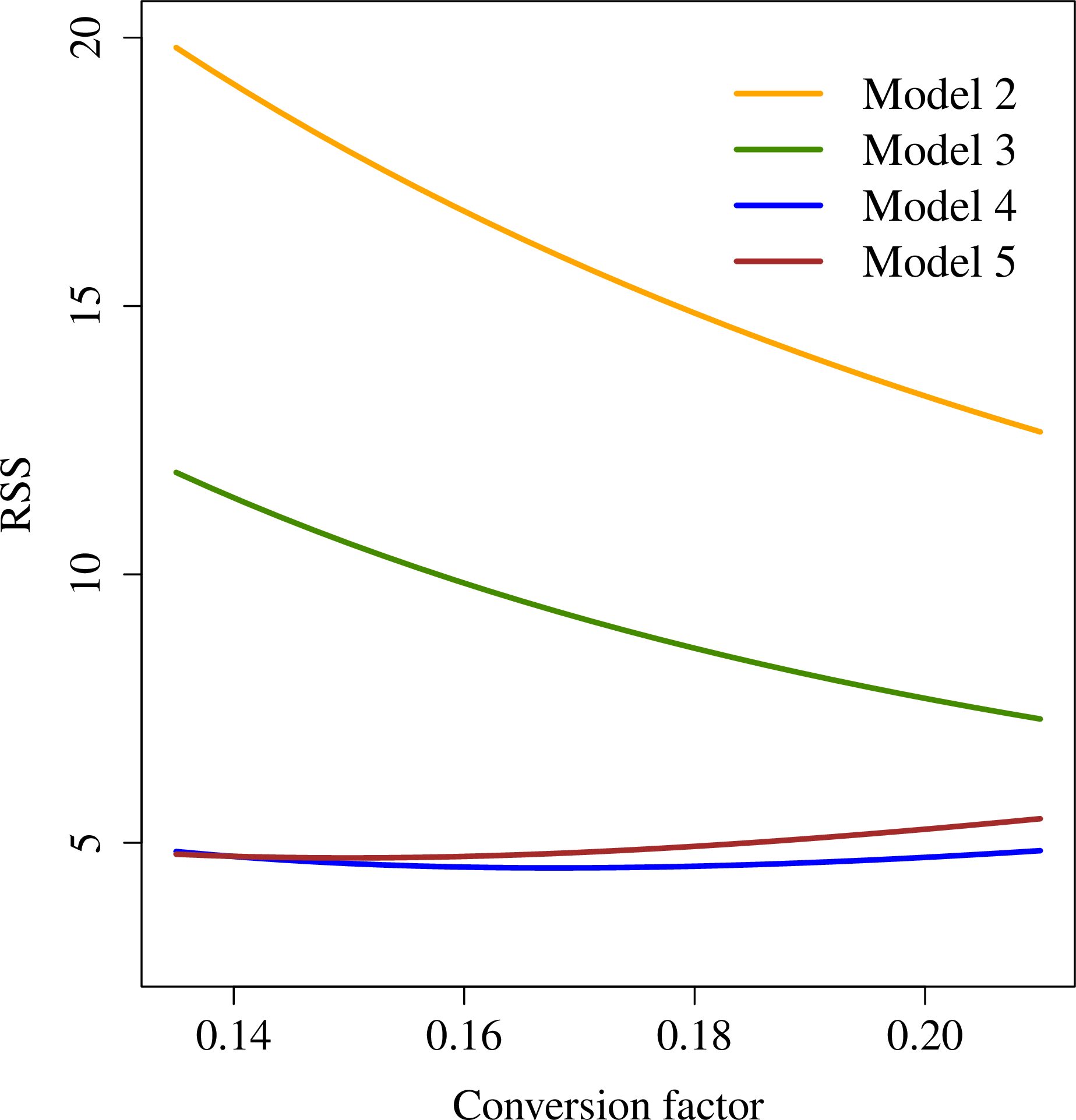

Supplement: S5 Fig — YFV-specific cells in the Akondy study were measured as % in lymphocytes and in the Marraco study as % in CD8 T cells, thus we converted the cell numbers from fitting Akondy data and model predictions using the following reasonings. Since 45–70% of PBMCs are T cells and about 30% of them are CD8 T cells, we used a conversion factor equal to 0.575 ⋅ 0.3 ≈ 0.17 in Fig 4. Plotting how this result depends on the conversion factor in a range of between 0.135 and 0.21 shows that Models 4 and 5 consistently show a better fit. Models colors are as in Fig 2: Model 2—orange, Model 3—green, Model 4—blue, Model 5—brown. The conversion factor only affects how well the predictions of the Models 1–5 will fit the Marraco data based on RSS. Thus, conversion factor will not affect the estimation of the CD8 T cell lifespans. (TIFF) [file pcbi.1009468.s005.tiff]

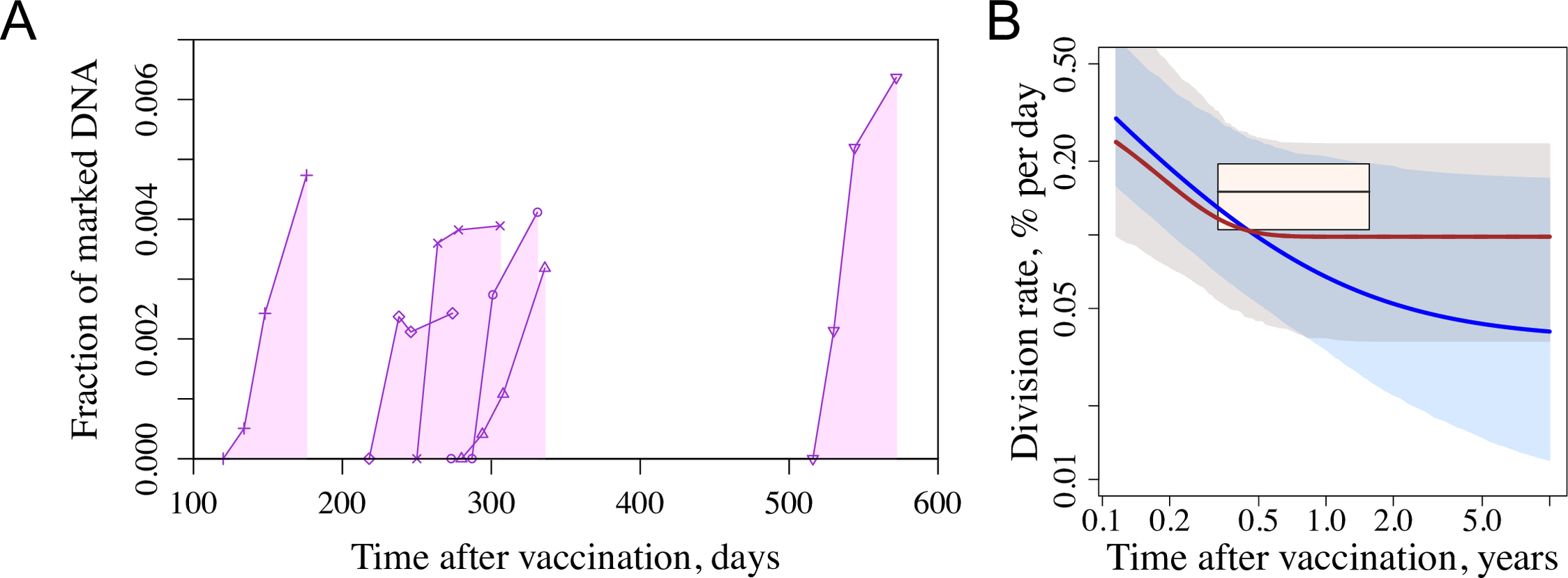

Supplement: S6 Fig — Panel A shows deuterium incorporation in six donors with heavy water consumption for 8 weeks starting at 4–19 months after YFV immunization (Study 3 in [24]). Data for different individuals are shown by different symbols. Pink shaded area indicates the period of heavy water consumption for each donor. Panel B shows how the predicted division rates (with their 95% confidence intervals (CI)) change with time. The asymptotic division rate for Model 4 (blue line) was estimated as 0.037% per day (95% CI 0.011–0.16) and for Model 5 (brown line) as 0.1% per day (95% CI 0.04139–0.236). The study analyzed the deuterium incorporation at the memory stage shown in Panel A [24], and the corresponding estimate for division rate, equal to 0.15±0.045% per day, is shown by a rectangle with 4–19 month length on the x-axis and standard deviation estimation on y-axis. (TIFF) [file pcbi.1009468.s006.tiff]

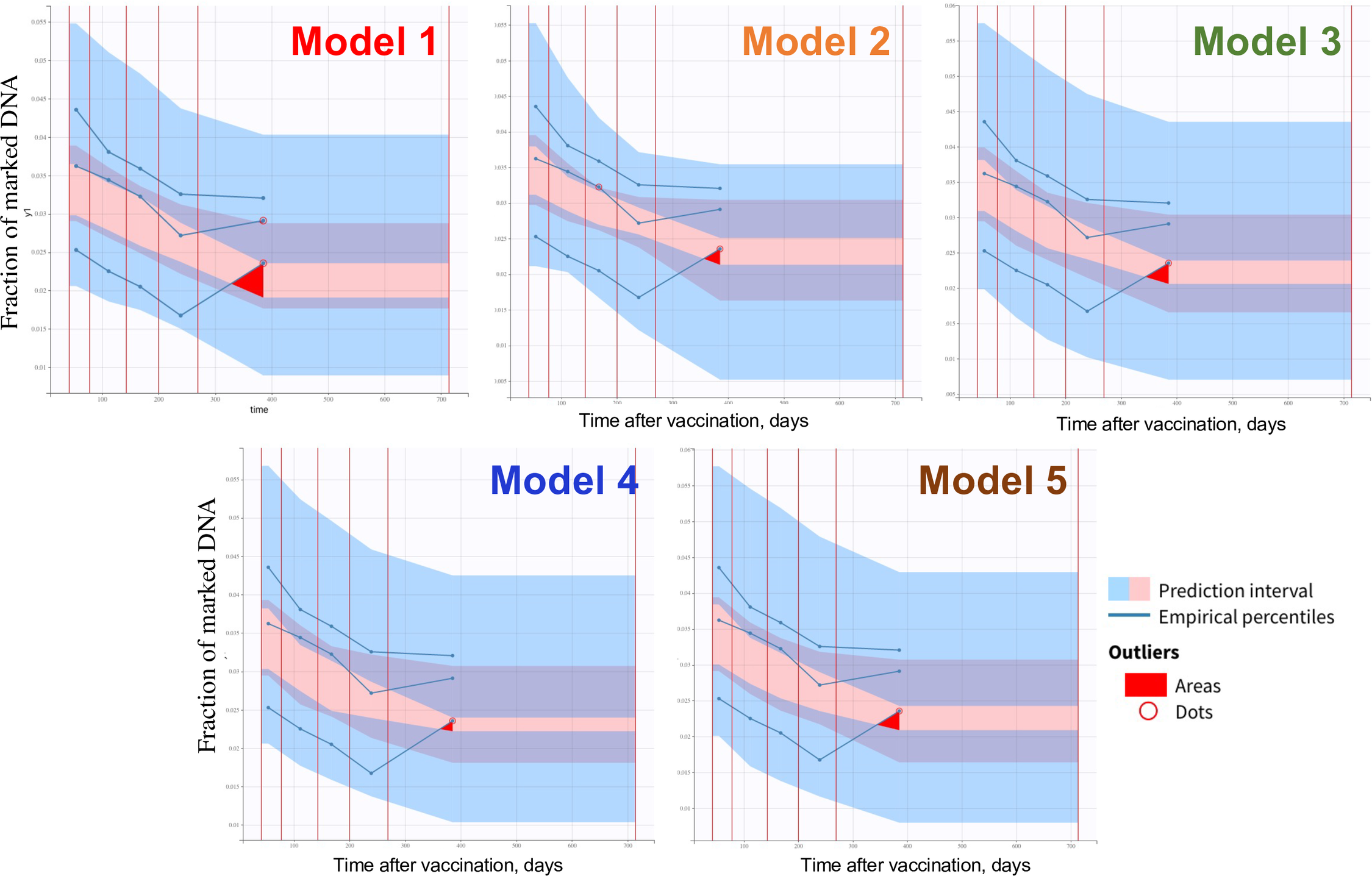

Supplement: S7 Fig — To additionally evaluate how well a given model can reproduce both the main trend and the variability in the data, we used Visual Predictive Check as implemented in Monolix. Different percentiles of the observed data (blue lines show 10th, 50th and 90th percentiles) are compared to 90% prediction intervals for those percentiles according to the models (blue shaded regions for 10th and 90th percentiles, pink for 50th percentile and purple for overlap). Red circles around a point indicate mismatch between the empirical percentiles and the model. Data are grouped together within bins of an independent variable (time). Binning criterion was “least-squares” (as implemented in Monolix) and bins are shown by red lines. VPC does not account for dropout. Most individuals dropped out before day 300 and hence VPC is not valid for the last bin shown in this figure. (TIFF) [file pcbi.1009468.s007.tiff]

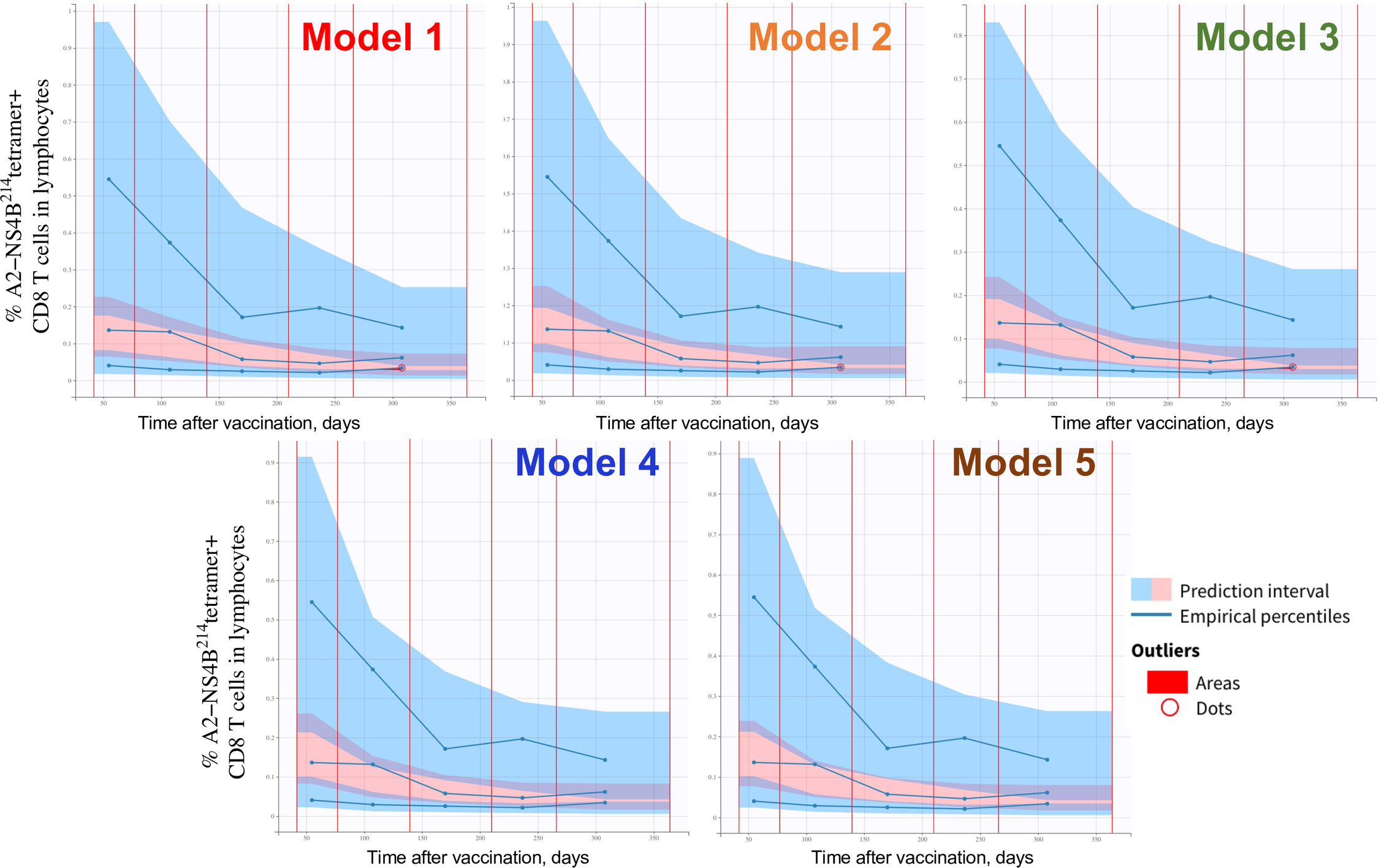

Supplement: S8 Fig — To additionally evaluate how well a given model can reproduce both the main trend and the variability in the data, we used Visual Predictive Check as implemented in Monolix. Different percentiles of the observed data (blue lines show 10th, 50th and 90th percentiles) are compared to 90% prediction intervals for those percentiles according to the models (blue shaded regions for 10th and 90th percentiles, pink for 50th percentile and purple for overlap). Red circles around a point indicate mismatch between the empirical percentiles and the model. Data are grouped together within bins of an independent variable (time). Binning criterion was “least-squares” (as implemented in Monolix) and bins are shown by red lines. (TIFF) [file pcbi.1009468.s008.tiff]

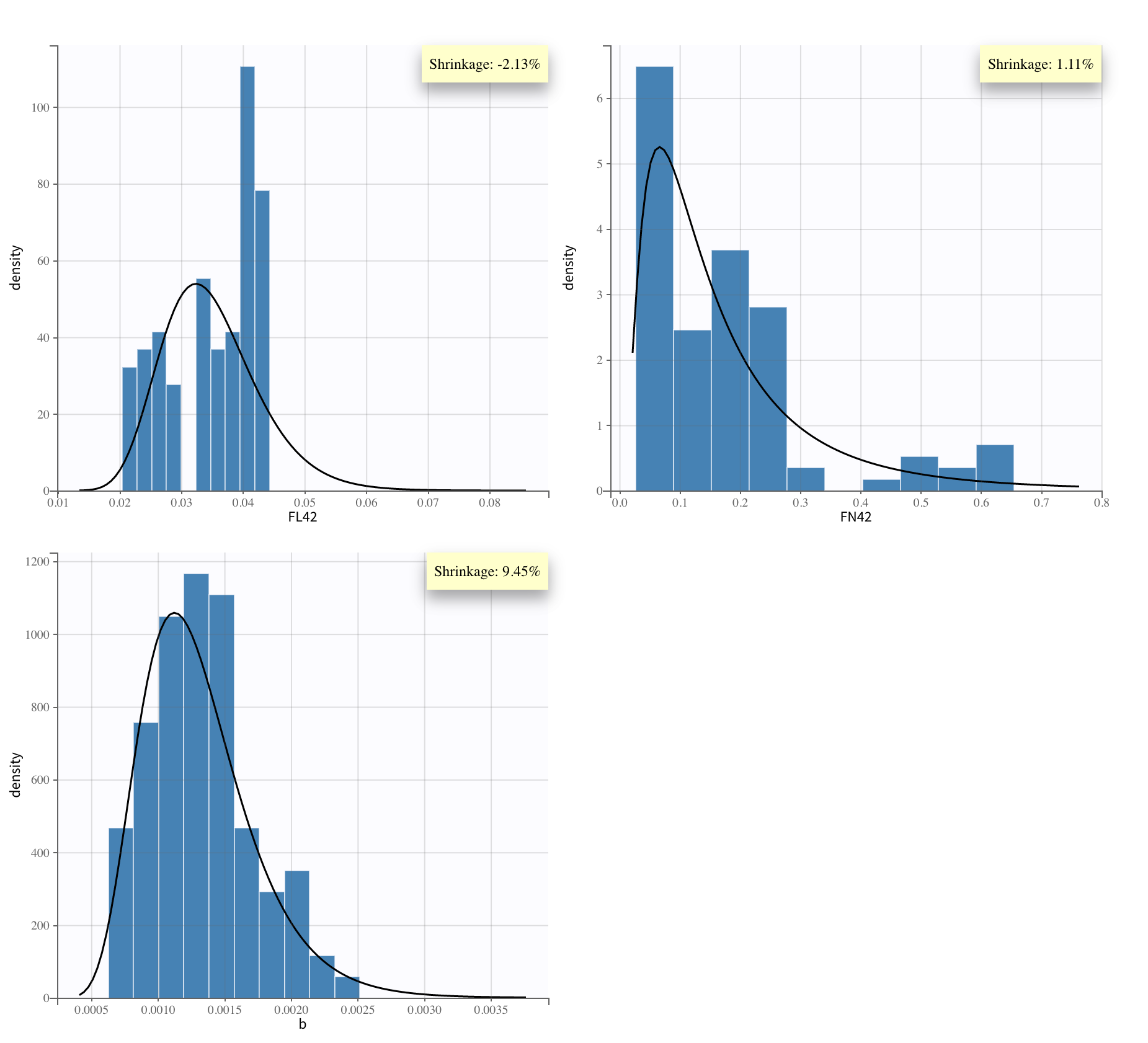

Supplement: S9 Fig — FL42 and FN42 are estimated fL and fN values at day 42, and b is a constant division rate. The death rate parameter d = 0.0067 per day (s.e. = 0.0007 per day; no random effects). Shrinkage for a model parameter η is calculated as 1−var(ηi^)/ω^2, where ηi^ are the posterior modes and ω^ is the estimated standard deviation of the random effects. (TIFF) [file pcbi.1009468.s009.tiff]

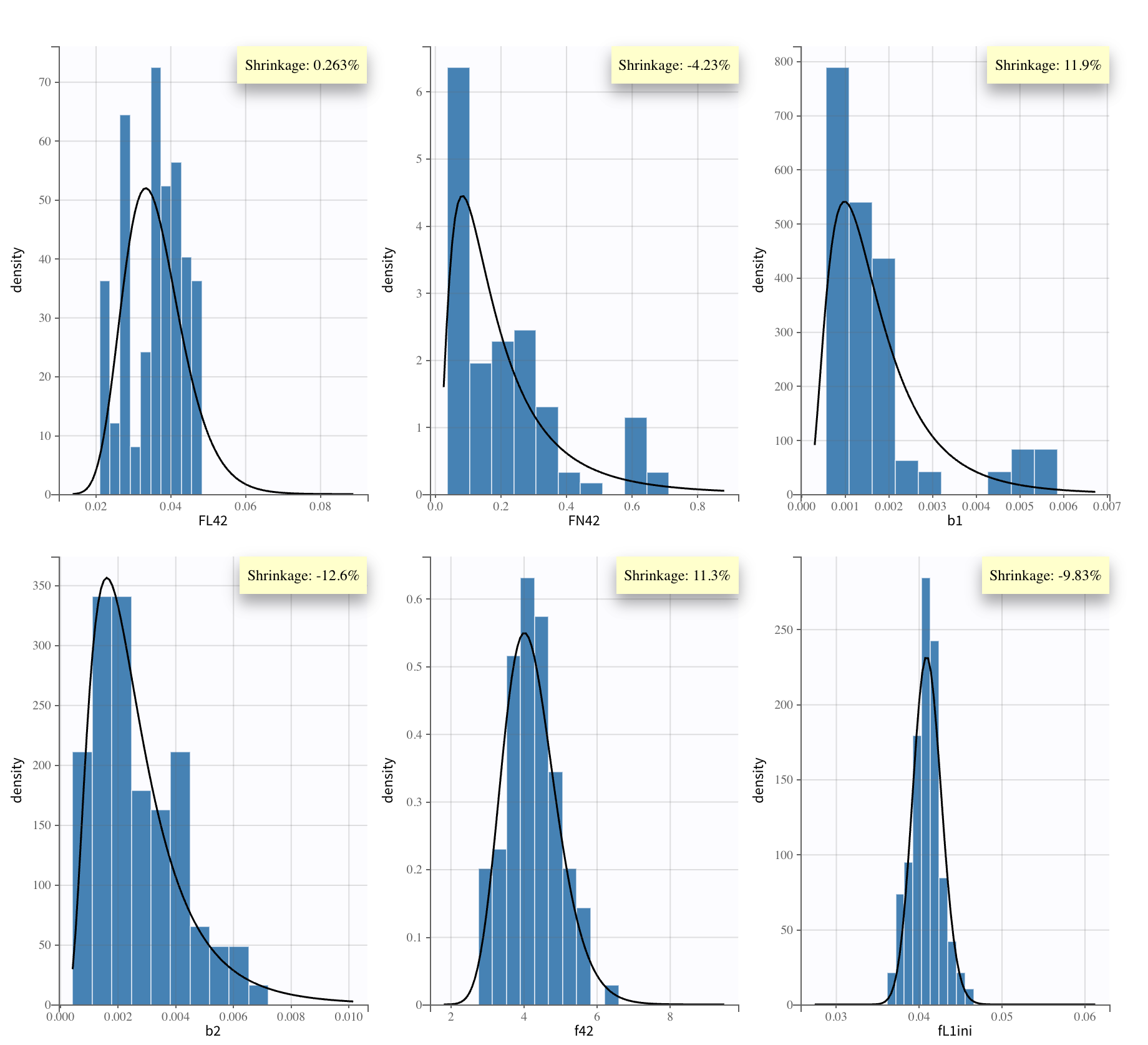

Supplement: S10 Fig — FL42 and FN42 are estimated fL and fN values at day 42. Parameters b1 and b2 are constant division rates for the two populations and other parameters are described in Methods. The death rate parameters are d1 = b1 and d2 = 0.0145 per day (s.e. = 0.0014 per day; no random effects). Shrinkage for a model parameter η is calculated as 1−var(ηi^)/ω^2, where ηi^ are the posterior modes and ω^ is the estimated standard deviation of the random effects. (TIFF) [file pcbi.1009468.s010.tiff]

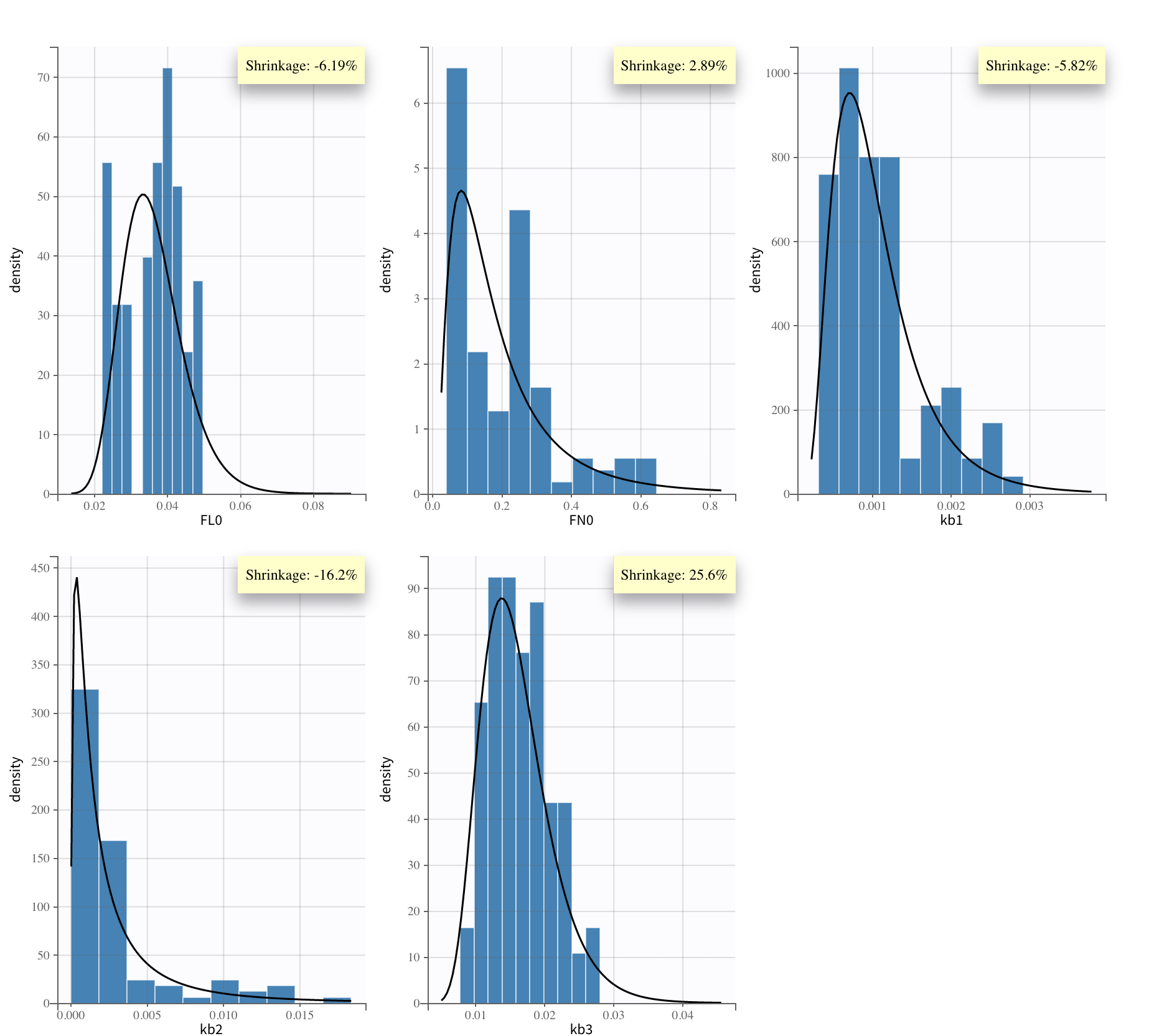

Supplement: S11 Fig — FL0 and FN0 are estimated fL and fN values at day 42. Parameters kb1, kb2, kb3 are estimates for the division rate in b(t) = kb1+kb2 exp(-kb3 t). The death rate parameters are d1 = kb1, d2 = 0.0129 per day (s.e. = 0.0017 per day) and d3 = 0.0048 (s.e. = 0.0004) in d(t) = d1+d2 exp(-d3 t) (no random effects for d2 and d3). Shrinkage for a model parameter η is calculated as 1−var(ηi^)/ω^2, where ηi^ are the posterior modes and ω^ is the estimated standard deviation of the random effects. (TIFF) [file pcbi.1009468.s011.tiff]

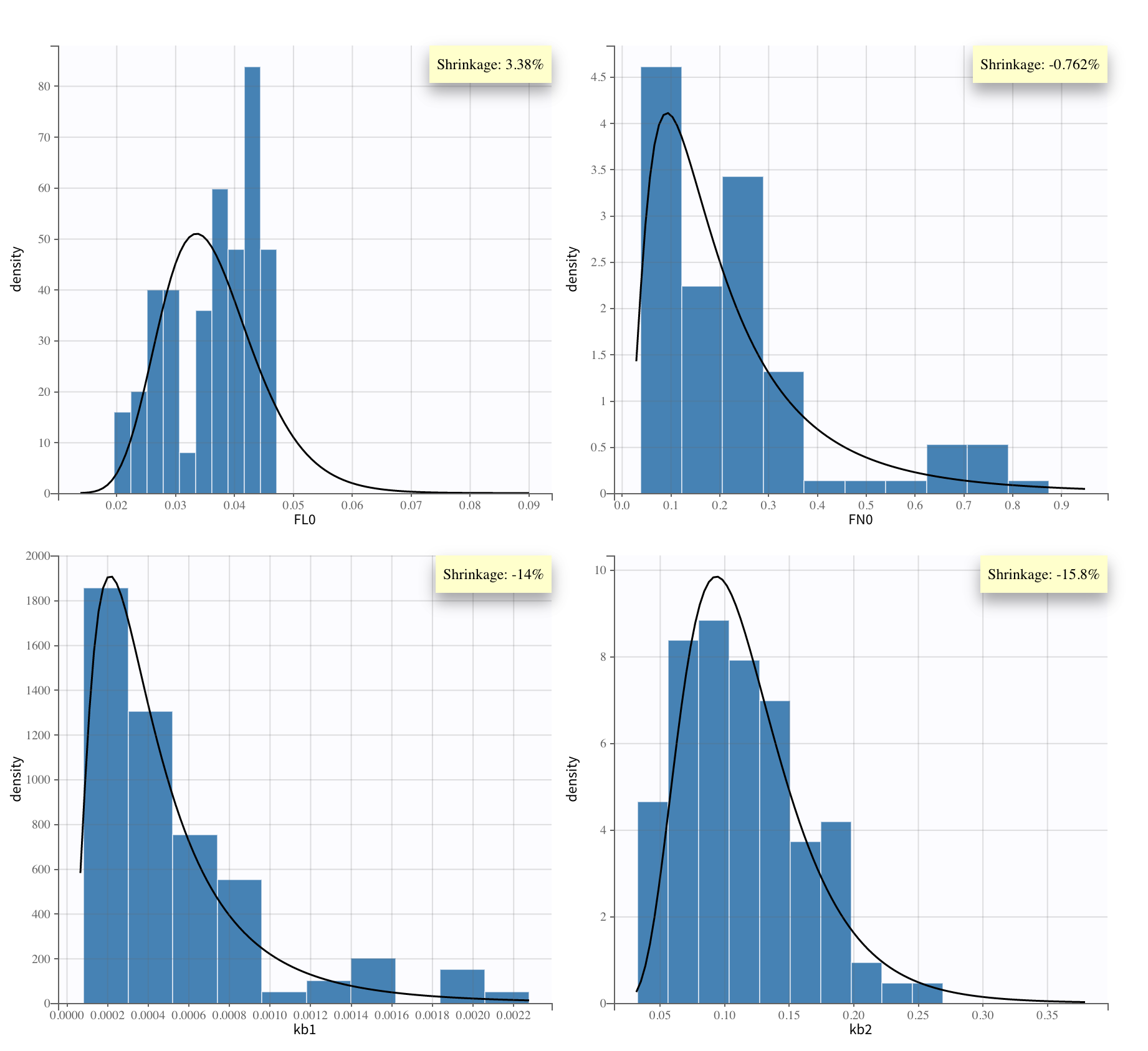

Supplement: S12 Fig — FL0 and FN0 are estimated fL and fN values at day 42. Parameters kb1, kb2 are estimates for the division rate in b(t) = kb1+kb2/t. The death rate parameters are d1 = kb1, d2 = 0.887 (s.e. = 0.0103) in d(t) = d1+d2/t (no random effects for d2). Shrinkage for a model parameter η is calculated as 1−var(ηi^)/ω^2, where ηi^ are the posterior modes and ω^ is the estimated standard deviation of the random effects. (TIFF) [file pcbi.1009468.s012.tiff]

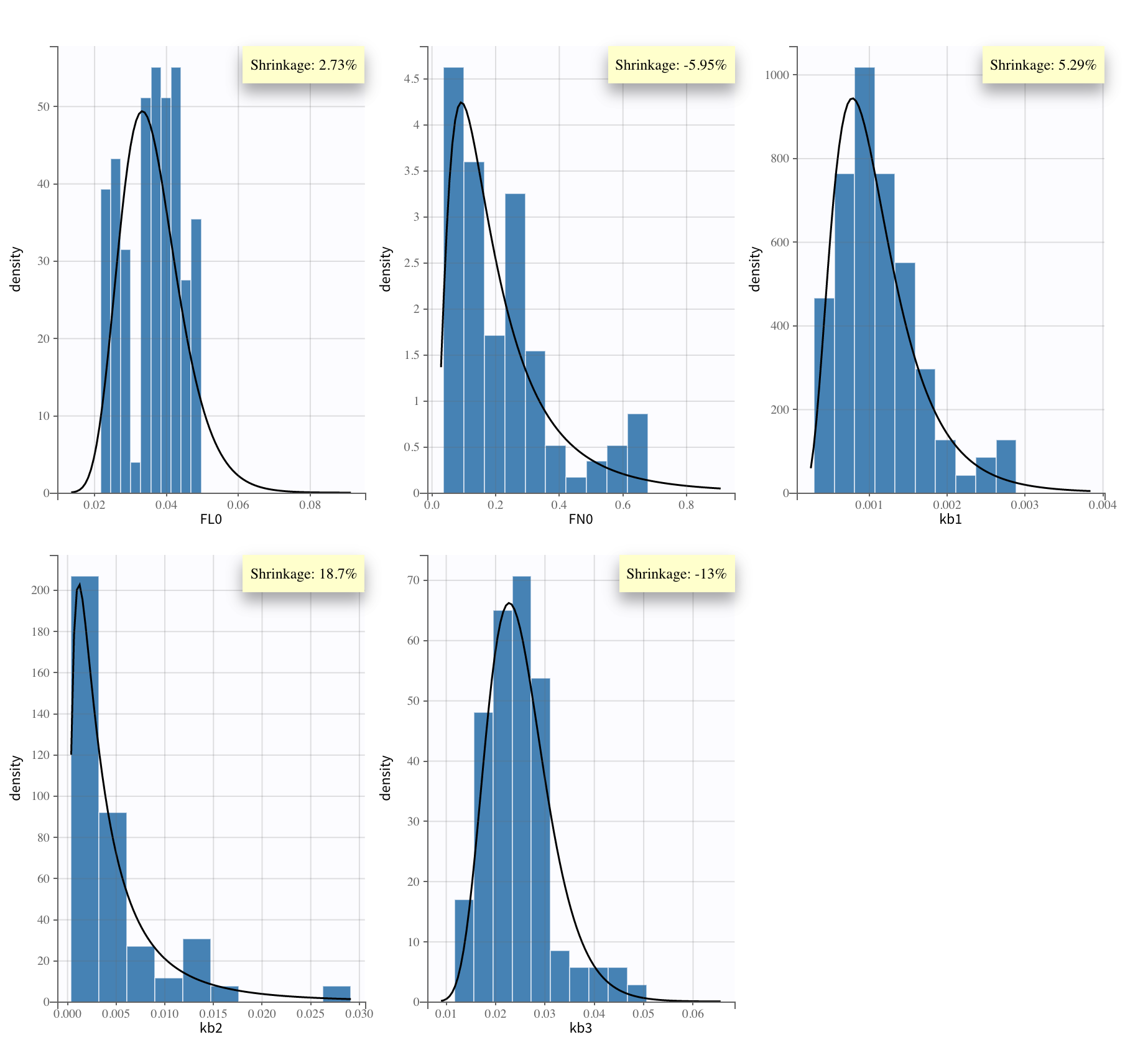

Supplement: S13 Fig — FL0 and FN0 are estimated fL and fN values at day 42. Parameters kb1, kb2, kb3 are estimates for the division rate in b(t) = kb1+kb2 exp(-kb3 t). The death rate parameters are d1 = kb1, d2 = 0.823 (s.e = 0.0186) in d(t) = d1+d2/t (no random effects for d2). Shrinkage for a model parameter η is calculated as 1−var(ηi^)/ω^2, where ηi^ are the posterior modes and ω^ is the estimated standard deviation of the random effects. (TIFF) [file pcbi.1009468.s013.tiff]
